# Combined associations of physical activity, diet quality and their changes over time with mortality: findings from the EPIC-Norfolk study, United Kingdom

**DOI:** 10.1186/s12916-024-03668-6

**Published:** 2024-10-14

**Authors:** Shayan Aryannezhad, Alexander Mok, Fumiaki Imamura, Nicholas J. Wareham, Soren Brage, Nita G. Forouhi

**Affiliations:** 1 MRC Epidemiology Unit, Institute of Metabolic Science, University of Cambridge School of Clinical Medicine, Cambridge, CB2 0QQ UK; 2 Singapore Institute for Clinical Sciences, Agency for Science, Technology and Research (A*STAR), Medical Drive, Brenner Centre for Molecular Medicine, Singapore, 117609 Republic of Singapore

**Keywords:** Physical activity, Diet quality, Mediterranean diet, Trajectories, Cardiovascular diseases mortality, Cancer mortality, Mortality, Prospective studies, Cohort studies

## Abstract

**Background:**

Physical activity (PA) and diet quality have each been shown to be inversely associated with mortality but their combined impact on longevity has been less explored, particularly when considering their changes over time. This study aimed to examine the separate and combined associations of PA, diet quality and their changes over time with mortality outcomes.

**Methods:**

A prospective cohort study was performed on 9349 adults aged 40 to 79 years from the population-based European Prospective Investigation into Cancer in Norfolk Study, with repeated measurements of PA and diet (from 1993 till 2004) and subsequent follow-up till 2022 (median follow-up 18.8 years). Validated questionnaires were used to derive physical activity energy expenditure (PAEE) as a proxy of total PA and adherence to the Mediterranean diet score (MDS, range 0–15 points) as an indicator of overall diet quality, and their changes over time (∆PAEE and ∆MDS). Cox regression models adjusted for potential confounders and mediators were used to estimate hazard ratios (HRs) and 95% CIs.

**Results:**

Over 149,681 person-years of follow-up, there were 3534 deaths. In adjusted models, for each 1-SD difference in baseline PAEE (4.64 kJ/kg/day), ∆PAEE (0.65 kJ/kg/day per year), baseline MDS (1.30 points) and ∆MDS (0.32 points per year), HRs (95% CI) for all-cause mortality were 0.90 (0.86 to 0.94), 0.89 (0.85 to 0.93), 0.95 (0.91 to 0.99) and 0.93 (0.90 to 0.97), respectively. Compared with participants with sustained low PAEE (< 5 kJ/kg/day) and low MDS (< 8.5 points), those with sustained high PAEE and high MDS had lower all-cause mortality (HR 0.78; 95% CI: 0.68–0.91), as did those who improved both PAEE and MDS (0.60; 0.44–0.82). There was no evidence of interaction between PA and diet quality exposures on mortality risk. Population impact estimates suggested that if all participants had maintained high levels of PA and diet quality consistently, cumulative adjusted mortality rate would have been 8.8% (95% CI: 2.4 to 15.3%) lower.

**Conclusions:**

These findings suggest that adopting and maintaining higher levels of PA and diet quality are associated with lower mortality. Significant public health benefits could be realised by enabling active living and healthy eating through adulthood.

**Supplementary Information:**

The online version contains supplementary material available at 10.1186/s12916-024-03668-6.

## Background

A large body of evidence from meta-analyses of prospective observational studies and randomised controlled trials has demonstrated that higher levels of physical activity (PA) and adherence to a Mediterranean-type diet (MED), separately, improve several cardiometabolic risk factors and are associated with lower incidence of cardiovascular disease (CVD), cancer and all-cause mortality [1–6]. Engaging in regular PA and adopting a Mediterranean-type dietary pattern in combination may therefore be optimal for reducing the burden and mortality of non-communicable diseases [7]; however, much of the evidence base comes from studies evaluating PA or MED independently, and their joint effect on health outcomes has been studied to a lesser extent simultaneously. To test the hypothesis that a high level of PA or alternatively a healthy diet can compensate for a lack of the other, it is essential to examine PA and diet as combined exposures in relation to health outcomes. Such investigations are important because of interrelated behavioural influences of PA and diet on each other [8].

Few studies have assessed the combined associations of PA and diet quality in relation to mortality. Prospective analyses of two Spanish cohorts have shown that being in the combined category of high PA and high adherence to MED is associated with 73% and 64% lower all-cause mortality, respectively, in each cohort [9, 10]. Similarly, a recent UK Biobank analysis found that the higher levels of PA and a higher diet quality in combination were associated with the lowest risk for all-cause mortality, CVD mortality and cancer mortality [11]. However, these estimates were based on a single (baseline only) measurement of both exposures. To capture the within-person variation of these health behaviours and the joint association of PA and diet quality over time, longitudinal analyses employing repeated exposure measures over several years are required. To our knowledge, only one study has done such an analysis. Using data from three phases of an Australian cohort study, Williamson and colleagues reported that sustained high PA and/or high MED over time was associated with lower all-cause mortality: 29% lower relative risk for high vs. low PA, 19% for high vs. low MED and a population attributable fraction of 18% for combined high PA and MED adherence [12]. However, longitudinal changes in PA or MED were not quantified and reported, nor were any specific trajectories identified that would allow the investigation of the associations with mortality for specific patterns of these two exposures. The formation of combined PA and diet trajectories in repeated measure analysis is more meaningful as it allows for the observation of patterns in health behaviours over time, providing a more accurate and dynamic understanding of their impacts.

Our aim was to examine the separate and combined associations of PA and MED with all-cause and cause-specific mortality accounting for distinct exposure trajectories and changes over time, using repeated assessment data from a population-based cohort of adults living in the UK.

## Methods

### Study design and population

**Fig. 1:**
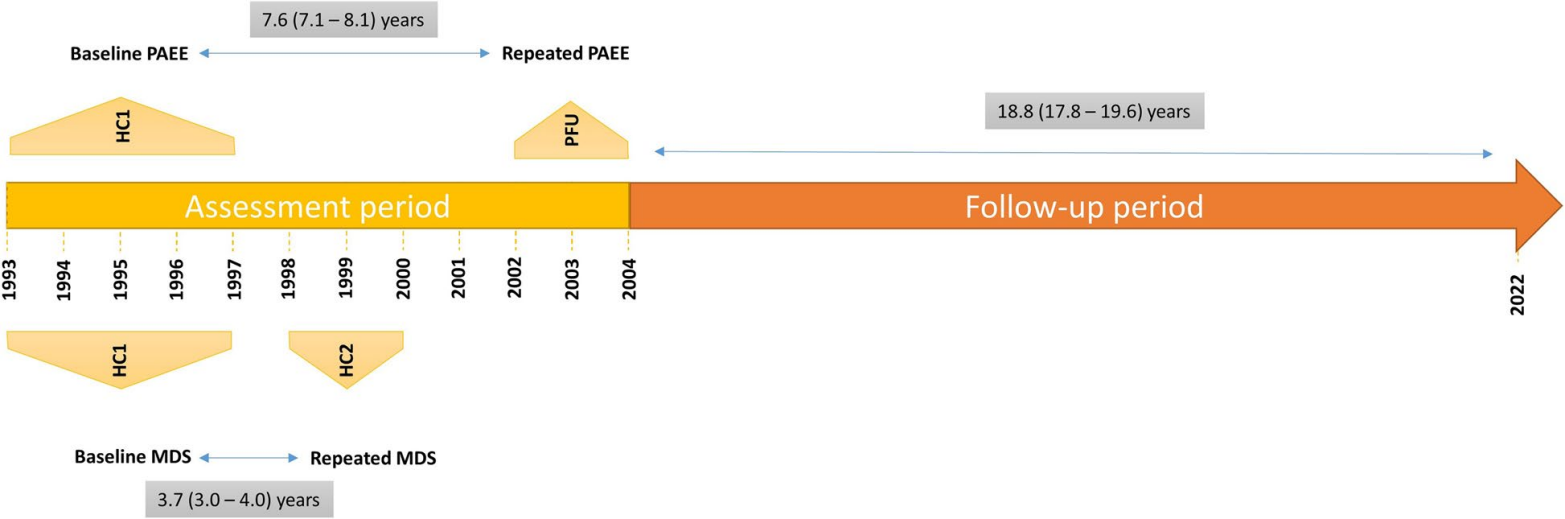
EPIC-Norfolk Study design and timeline

The European Prospective Investigation into Cancer in Norfolk (EPIC-Norfolk) Study is a population based cohort study of 25,639 men and women aged 40 to 79 years resident in Norfolk, UK and recruited from 1993 to 1997. Further data were collected prospectively at different health checks (HC) at clinics and postal follow-ups (PFU) using mailed questionnaires. Details of the study design have been previously described [13]. For the current investigation, the study data were divided into two different periods: an exposure assessment period from 1993 to 2004 and a subsequent follow-up period for mortality outcomes up until 2022 (Fig. 1). The first assessment of both PA and diet was at the HC1 (1993 to 1997). A diet assessment was repeated at HC2 (1998–2000), while repeated PA assessment was done using a postal questionnaire at PFU (2002 to 2004). We excluded participants who did not have assessments for both PA and diet at HC1 (*n* = 1947), those who did not attend HC2 or without assessment of diet at HC2 (*n* = 12,227), as well as those without assessments for PA at PFU (*n* = 2116); leaving us with the analytical sample (*n* = 9349) consisting of those who had repeated measures of both health behaviours during the assessment period (Additional file 1: Fig. S1). Outcome ascertainment was documented in the follow-up period. For a subsample of the population (*n* = 5878), a third assessment of both diet and PA occurred at HC3 (2005–2011), data from whom were included in sensitivity analyses.

### Assessment of PA

At both baseline and repeat assessments, participants’ habitual PA was assessed using a questionnaire which queried habitual PA of past 12 months. Briefly, the questionnaire asked about occupational physical activity (OPA) as well as leisure-time physical activity (LTPA). Participants self-categorised their OPA as either unemployed, sedentary, standing, physical work or heavy manual work. For LTPA, participants answered questions enquiring about time spent on walking, cycling, gardening, housework and exercises in summer and winter separately. This questionnaire has been validated against and calibrated to physical activity energy expenditure (PAEE, in units of kJ/day/kg) assessed using individually calibrated combined heart rate and movement sensing as previously described [14–16]. We have previously shown that PAEE estimated using this method predicts mortality among EPIC-Norfolk participants [16].

### Assessment of diet

Participants’ habitual diet over the past year was assessed using a 130-item semi-quantitative food frequency questionnaire (FFQ). In a subsample of EPIC-Norfolk, validity of this FFQ was assessed previously for major foods and nutrients against 16-day weighed dietary records, 24-h recall and specific biomarkers [17–19]. To assess overall diet quality by adherence to a predefined healthy dietary pattern, the Mediterranean diet score (MDS) based on the Mediterranean dietary pyramid was used [20, 21]. In brief, the MDS assigned a continuous value ranging from 0 to 1 to each of the following 14 dietary components based on the degree of achieving each recommended intake for vegetables, legumes, fruits, nuts, cereals, dairy, fish, red meat, processed meat, white meat, eggs, potatoes, sweets and alcohol. The fifteenth component was olive oil consumption, with a binary score of 0 for non-consumers and 1 for consumers. The MDS could range from 0 to 15, with 15 being the highest diet quality. The MDS has been shown to predict CVD incidence and mortality in this population [21].

### Assessment of covariates

At the two clinic visits (HC1 and HC2), trained research nurses obtained measures of height and weight using standardised protocols from which body mass index (BMI) in kg/m^2^ was computed [22]. Systolic and diastolic blood pressure (SBP and DBP) were assessed while seated. Serum triglycerides (TG), low-density lipoprotein cholesterol (LDL-C) and high-density lipoprotein cholesterol (HDL-C) were measured in non-fasted blood samples [22]. Participants self-reported their age, sex, smoking status, education level and employment status. For this study, smoking status was categorised into three levels (current smokers; former smokers; never smokers); education level was categorised into two levels (General Certificate of Education [GCE] Ordinary Level or below; GCE Advanced Level, bachelor’s degree and above) and employment type was categorised into three levels (unemployed to semi-skilled work; skilled workers; managers and professionals). Medication use, comorbidities and family history of chronic diseases were self-reported. Additionally, data from hospital episode statistics were used to ascertain updated medical histories on prevalent diabetes, CVD and cancer for the entire exposure assessment period.

### Mortality ascertainment

Vital status was assessed in the study follow-up period via record linkage with the Office of National Statistics, from the date of last repeated assessment of PA or diet until the study censor date (31st of March 2022). Causes of death were confirmed by death certificates, coded according to international classification of diseases versions 9 and 10 (ICD-9 and ICD-10). Cardiovascular disease (CVD) mortality and cancer mortality were defined by ICD-9 codes 400–438 or ICD-10 I10–I79 and ICD-9 codes 140–208 or ICD-10 C00–C97, respectively. CVD subtypes (ischemic heart disease [IHD] and stroke) and site-specific cancers (lung, prostate, breast, oesophagus, stomach and colorectal) were also ascertained using ICD-9 and ICD-10 codes.

### Statistical analysis

Paired *t*-tests were used to compare means in the same population (repeated measurements of the analytical sample) and unpaired *t*-tests for independent populations (included vs. excluded sample). Proportions were compared using chi-square test. Follow-up person-years were calculated from the last repeated assessment of PA or diet to the time of death, or the censor date (31/03/2022). Cox proportional hazard regression models were fitted to estimate hazard ratios (HR) and 95% confidence intervals (95% CI) for the prospective associations between PA, diet and mortality. Longitudinal within-person changes in PAEE (∆PAEE) and MDS (∆MDS) were calculated by subtracting the corresponding value at the baseline (HC1) exposure values from the value at the repeated assessment (Fig. 1), divided by the time (in years). These change variables were expressed in units of kJ/kg/day per year for ∆PAEE and score points per year for ∆MDS. In the survival analysis, four mutually adjusted exposure variables (baseline PAEE, ∆PAEE, baseline MDS and ∆MDS) were included simultaneously as continuous variables into the Cox models. Proportional hazards assumption was tested using Schoenfeld residuals. Linear relationships between the log-hazard and the continuous covariates were assessed visually by plotting Martingale residuals. Multicollinearity was investigated by assessing correlation matrix of coefficients of the Cox models.

To investigate the specific trajectory combinations of PA and diet over time, we cross-classified both exposures at both baseline and repeated assessments, creating 16 different trajectory groups (G1 to G16). Specifically, these were assigned according to dichotomous achievement of a healthy PA behaviour (PAEE ≥ 5 kJ/kg/day, equivalent to 150 min/week of moderate-to-vigorous PA) and a healthy overall diet quality (above median of MDS points in the cohort). Groups were formed according to having a high (H) or low (L) level of baseline PA level, baseline MDS level, repeated PA level and repeated MDS level as follows:

- G1: LLLL (low PA and low MDS at baseline and at repeated assessments)
- G2: LLHL (low PA and low MDS at baseline, high PA and low MDS at repeated assessments)
- G3: LLLH (low PA and low MDS at baseline, low PA and high MDS at repeated assessments)
- G4: LLHH (low PA and low MDS at baseline, high PA and high MDS at repeated assessments)
- G5: HLLL (high PA and low MDS at baseline, low PA and low MDS at repeated assessments)
- G6: HLHL (high PA and low MDS at baseline and at repeated assessments)
- G7: HLLH (high PA and low MDS at baseline, low PA and high MDS at repeated assessments)
- G8: HLHH (high PA and low MDS at baseline, high PA and high MDS at repeated assessments)
- G9: LHLL (low PA and high MDS at baseline, low PA and low MDS at repeated assessments)
- G10: LHHL (low PA and high MDS at baseline, high PA and low MDS at repeated assessments)
- G11: LHLH (low PA and high MDS at baseline and at repeated assessments)
- G12: LHHH (low PA and high MDS at baseline, high PA and high MDS at repeated assessments)
- G13: HHLL (high PA and high MDS at baseline, low PA and low MDS at repeated assessments)
- G14: HHHL (high PA and high MDS at baseline, high PA and low MDS at repeated assessments)
- G15: HHLH (high PA and high MDS at baseline, low PA and high MDS at repeated assessments)
- G16: HHHH (high PA and high MDS at baseline and at repeated assessments)

In the Cox proportional hazards regression analysis, the 16 trajectory groups were entered as categorical variables, keeping the group with low PA and low diet quality at both baseline and repeated assessments (G1) as the reference. We also conducted stratified analyses by the four PA-diet categories at baseline. In each stratum, the reference group was set as the group that maintained the baseline levels of PAEE and MDS over the assessment period.

The modelling strategy was as follows. Model 1 adjusted for sex and age (minimally adjusted). Model 2 additionally included education, employment and time-updated variables for smoking (socioeconomic and behavioural confounders). Model 3 was further adjusted for family history of myocardial infarction and diabetes mellitus [DM], time-updated variables for prevalent diseases (DM, cancer, CVD) and the use of anti-hypertensive medication (history of diseases as confounders). Finally, model 4 additionally included time-updated variables for BMI, SBP, DBP, TG, LDL-C, HDL-C and total energy intake (potential mediators). Missing covariates were imputed using a multiple imputation technique with chained equations, in which the distribution of the observed available variables (age, sex, MDS, PAEE) was used to estimate plausible values for the missing data [23].

To examine possible synergistic or compensatory effects between PA, diet quality and their changes over time, tests of interaction between different combinations of these exposures for the all-cause mortality outcome were performed on both multiplicative and additive scales (information in supplementary materials) [24]. Stratified analyses were performed to test for potential interaction by age, sex, BMI, smoking and pre-existing comorbidities (any of the following: cancer, cardiovascular disease, diabetes or hypertension). Four interaction terms, each corresponding to one of the exposures and the stratifying variable, were simultaneously included in a regression model. The *P* values for these interaction terms were reported to evaluate the statistical significance of interaction by subgroup. To assess the population impact, we first calculated adjusted mortality rates for each trajectory group (G1 to G16) using multivariable exponential regression (from model 4). Next, we estimated the differences that could have been observed in the adjusted number of deaths in each trajectory group under two counterfactual scenarios. Scenario 1 calculated adjusted mortality if the entire population had had sustained low levels of PA and diet quality throughout the assessment period (i.e. applying the adjusted mortality rate of G1 to all other trajectories). Scenario 2 calculated adjusted mortality if the entire population had had sustained high levels of PA and diet quality throughout the assessment period (i.e. applying the adjusted mortality rate of G16 to all other trajectories). We computed the person-time distribution by considering a new survival probability for each trajectory group according to each scenario. The adjusted number of deaths under scenario 1 minus the adjusted observed number of deaths was calculated to estimate how many deaths were theoretically averted because of the observed PAEE and MDS trajectories. The adjusted observed number of deaths minus that under scenario 2 was calculated to estimate how many deaths would have been averted if all the participants sustained high PAEE and high MDS over the assessment period. Accordingly, population impact estimates were computed by summing up the differences in the number of deaths across all exposure groups for each of the two scenarios, expressed relative to the total adjusted number of deaths. We calculated the 95% CI for these estimates using a bootstrapping approach. Cumulative adjusted mortality rate of the population during two decades of follow-up was also calculated under each scenario.

A series of ancillary analyses were performed to examine the robustness of findings. The Cox regression models were repeated in complete-case analyses, without imputation of missing covariates. Two alternative approaches were implemented to investigate repeated assessments of PA and diet. First, cumulative average scores of PAEE and MDS were introduced as two mutually adjusted exposures in the Cox regression models to represent overall long-term PA and diet quality. Second, in the subsample of participants with available data, we extended the end of the assessment period to HC3 (2005–2011); relevant ∆PAEE and ∆MDS variables were re-calculated using baseline together with this later assessment covering a longer exposure period (and shorter follow-up for mortality). Additionally, to reduce the possibility of reverse causation, we excluded individuals who died within 2 years of the last repeated assessment of PA and diet. Fractional polynomial approach was conducted to allow flexible, non-linear assessment of the HR (95% CI) of exposures for all-cause mortality and create dose–response curves. To address the possibility that the dichotomisation approach for creating the joint trajectories may oversimplify the true underlying population differences in PA and diet, a further analysis was conducted using trajectories based on three exposure levels of PA and diet at baseline and repeat assessments, yielding 9 trajectory groups for each health behaviour (controlling for the other). All analyses were performed using Stata software version 16.

## Results

### Participant characteristics

During the exposure assessment period (from 1993 to 2004), the median (interquartile range) time elapsed between the baseline and repeated measurement were 7.6 years (7.1–8.1) for PA and 3.7 years (3.0–4.0) for diet and other covariates (Table 1 and Fig. 1). At baseline, the 9349 participants had a mean age of 58.3 (SD 8.7) and 58.6% were women. The mean baseline PAEE was 6.04 (SD 4.64) kJ/kg/day, and mean baseline MDS was 8.55 (SD 1.30) points, with PAEE decreasing slightly and MDS increasing slightly over time. Between the two assessments, the mean BMI, SBP, TG, HDL-C and alcohol intake increased, but the mean DBP, total cholesterol and LDL-C decreased. The prevalence of DM, CVD, cancer and the use of statins and anti-hypertensive medications increased over time, whereas the number of current smokers declined. Compared to those still alive, participants who died by the end of the follow-up were older at baseline, with higher levels of cardiometabolic risk factors and comorbidities and lower levels of PAEE and MDS (Additional file 1: Table S1). Compared to the analytical sample, the excluded sample was older, had a higher proportion of men, had a higher level of metabolic risk factors, had a higher prevalence of comorbidities and medication use at baseline and lower levels of PAEE and MDS (Additional file 1: Table S2).

**Table 1:** Participant characteristics at baseline and repeated assessments of the EPIC-Norfolk Study (*n* = 9349)^a^

Characteristic	Baseline assessment (1993–1997)	Repeated assessment (1998–2004)^a^
Demographics		
Age (years)	58.3 (8.7)	61.5 (8.8)
Women (%)	58.6	
Education: GCSE/O level equivalent or below (%)	41.7	
Occupation: unemployed to semi-skilled work (%)	15.5	
Occupation: skilled workers (%)	36.9	
Occupation: managers and professionals (%)	47.5	
Metabolic risk factors		
Body mass index (kg/m^2^)	25.9 (3.7)	26.5 (3.9)
Systolic blood pressure (mmHg)	134.5 (17.7)	135.0 (17.8)
Diastolic blood pressure (mmHg)	82.2 (10.9)	82.1 (11.0)
Triglycerides (mmol/L)	1.73 (1.05)	1.84 (1.07)
Total cholesterol (mmol/L)	6.15 (1.15)	6.06 (1.15)
HDL-cholesterol (mmol/L)	1.43 (0.43)	1.51 (0.46)
LDL-cholesterol (mmol/L)	3.95 (1.03)	3.77 (1.04)
Prevalent diseases, medication and family history		
Diabetes mellitus (%)	1.4	2.9†
Cardiovascular diseases (%)	0.1	11.8†
Cancer (%)	5.1	9.7†
Statins (%)	0.8	3.8
Anti-hypertensive drugs (%)	13.9	20.8
Family history of diabetes mellitus (%)	13.6	
Family history of myocardial infarction (%)	37.2	
Health behaviours		
PAEE (kJ/kg/day)	6.04 (4.64)	4.99 (4.64)†
ΔPAEE (kJ/kg/day per year)	–	− 0.13 (0.65)†
MDS points	8.55 (1.30)	8.66 (1.29)
ΔMDS (points per year)	–	0.03 (0.33)
Current smoker (%)	7.9	6.7
Former smoker (%)	39.6	40.9
Energy intake (kcal/day)	2059 (583)	1964 (559)
Alcohol (g/day)	8.5 (12.4)	8.8 (12.6)

Data are presented as mean (SD) or %

*HDL* High-density lipoprotein, *LDL* Low-density lipoprotein, *PAEE* Physical activity energy expenditure, *ΔPAEE* Over time changes in PAEE, *MDS* Mediterranean diet score, *ΔMDS* Over time changes in MDS

*P *value ≤ 0.05 in all paired comparison between baseline assessment and repeated assessment, except for diastolic blood pressure

^a^For all variables, except those indicated by a dagger symbol (†), the repeated assessment period is health check 2 (HC2: 1998–2000). For the variables indicated by the dagger symbol, the repeated assessment period is the time of postal follow-up (PFU: 2002–2004)

### Associations of baseline and changes in PA and diet quality with mortality

There were 3534 total deaths (from all causes), including 2978 CVD deaths and 1794 cancer deaths, during 149,681 person-years of follow-up (median [IQR] of 18.8 years [17.9–19.6]). In the Cox models for the main four exposures, the proportional hazards assumption was met, linear relationships with log-hazards were observed, and multicollinearity was not detected. Table 2 shows the associations of mutually adjusted exposures with all-cause mortality, CVD mortality and cancer mortality. The magnitude and significance of the associations were consistent for all four continuous exposure variables across all models. In the most adjusted model (model 4), for each 1-SD higher baseline PAEE (4.64 kJ/kg/day), ∆PAEE (0.65 kJ/kg/day per year), baseline MDS (1.30 points) and ∆MDS (0.33 points per year), the HRs (95% CI) for all-cause mortality were 0.90 (0.86 to 0.94), 0.89 (0.85 to 0.93), 0.95 (0.91 to 0.99) and 0.93 (0.90 to 0.97), respectively. All baseline and delta exposures were also inversely associated with CVD mortality (HRs ranging from 0.90 to 0.94); this was also the pattern of association for cancer mortality (HRs ranging from 0.91 to 0.98). Sensitivity analysis using the complete case analysis (excluding participants with missing covariates) (Additional file 1: Table S3) yielded similar results. Results for mortality due to CVD subtypes (IHD and stroke) and site-specific cancers (lung, prostate, breast, GI) were generally consistent with the overall associations for CVD and cancer mortality (Additional file 1: Table S4). No significant interactions were found in either multiplicative or additive scales, for any combination of exposures on all-cause mortality (Additional file 1: Table S5). Findings remained consistent when using cumulative averaged PAEE and MDS as indicators of long-term PA and diet behaviours (Additional file 1: Table S6) and in all sensitivity analyses, including when exposure assessment period was extended to HC3 (Additional file 1: Fig. S2 and Additional file 1: Table S7), when the dose–response associations with all-cause mortality were assessed using non-linear modelling (Additional file 1: Fig. S3) and when participants who died within 2 years of the last repeated assessment were excluded (Additional file 1: Table S8).

**Table 2:** Associations of mutually adjusted exposures with mortality outcomes in the EPIC-Norfolk Study^a^

Outcome	Exposures^b^	Model 1 HR (95% CI)	Model 2 HR (95% CI)	Model 3 HR (95% CI)	Model 4 HR (95% CI)
**All-cause mortality (3534 deaths)**	Baseline PAEE	0.87 (0.83 to 0.91)	0.88 (0.84 to 0.92)	0.90 (0.86 to 0.94)	0.90 (0.86 to 0.94)
	ΔPAEE	0.87 (0.83 to 0.91)	0.88 (0.84 to 0.92)	0.89 (0.85 to 0.93)	0.89 (0.85 to 0.93)
	Baseline MDS	0.92 (0.88 to 0.95)	0.94 (0.90 to 0.98)	0.94 (0.90 to 0.97)	0.95 (0.91 to 0.99)
	ΔMDS	0.92 (0.88 to 0.95)	0.93 (0.90 to 0.97)	0.93 (0.90 to 0.97)	0.93 (0.90 to 0.97)
**CVD mortality (2978 deaths)**	Baseline PAEE	0.87 (0.83 to 0.91)	0.88 (0.84 to 0.93)	0.91 (0.87 to 0.96)	0.91 (0.87 to 0.95)
	ΔPAEE	0.87 (0.83 to 0.92)	0.88 (0.84 to 0.93)	0.90 (0.86 to 0.95)	0.90 (0.86 to 0.95)
	Baseline MDS	0.91 (0.87 to 0.95)	0.93 (0.89 to 0.97)	0.92 (0.89 to 0.97)	0.94 (0.90 to 0.99)
	ΔMDS	0.92 (0.88 to 0.96)	0.93 (0.89 to 0.97)	0.93 (0.89 to 0.97)	0.94 (0.90 to 0.98)
**Cancer mortality (1794 deaths)**	Baseline PAEE	0.89 (0.84 to 0.95)	0.90 (0.85 to 0.96)	0.92 (0.87 to 0.98)	0.92 (0.87 to 0.98)
	ΔPAEE	0.88 (0.83 to 0.93)	0.89 (0.83 to 0.94)	0.91 (0.86 to 0.97)	0.91 (0.86 to 0.97)
	Baseline MDS	0.95 (0.90 to 1.00)	0.97 (0.92 to 1.03)	0.97 (0.92 to 1.03)	0.98 (0.93 to 1.04)
	ΔMDS	0.93 (0.88 to 0.98)	0.94 (0.89 to 0.99)	0.94 (0.89 to 0.99)	0.94 (0.88 to 0.99)

*CI* Confidence interval, *CVD* Cardiovascular diseases, *EPIC* European Prospective Investigation of Cancer and nutrition, *HR* Hazard ratio, *MDS* Mediterranean diet score, *PAEE* Physical activity energy expenditure, *SD* Standard deviation

^a^HR per SD difference in each exposure are presented. *n* = 9349, person-years = 149,681. All coefficients are mutually adjusted for the four primary exposures. Covariates in the models: Model 1: sex, age. Model 2: variables in Model 1 + education, employment, and time updated variables for smoking. Model 3: variables in Model 2 + family history of myocardial infarction, family history of diabetes mellitus, time updated variables for prevalent diabetes mellitus, prevalent cancer, prevalent cardiovascular diseases, and anti-hypertensive medication. Model 4: variables in Model 3 + time updated variables for body-mass index, systolic blood pressure, diastolic blood pressure, triglycerides, low-density lipoprotein cholesterol, high-density lipoprotein cholesterol, and total energy intake. ^b^SD increment in baseline PAEE equals 4.64 kJ/kg/day, in ΔPAEE equals 0.65 kJ/kg/day per year, in baseline MDS equals 1.30 points, and in ΔMDS equals 0.33 points per year

### Joint associations of different trajectories of PA and diet quality with mortality

**Fig. 2:**
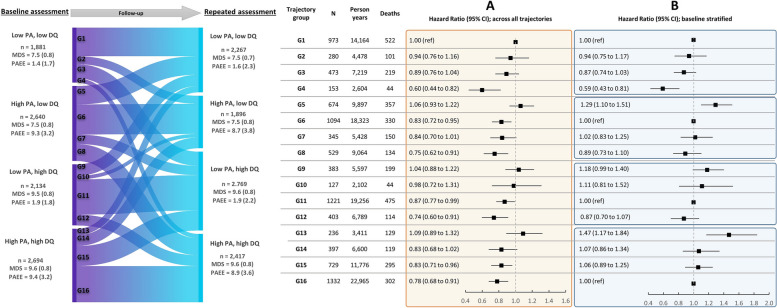
Joint associations of trajectory patterns of PA and diet with all-cause mortality in the EPIC-Norfolk Study. **A** Comparison across all trajectories together (G1 as reference); **B** Stratified analysis by baseline exposure level (stable behaviours as reference groups). Analysis is based on model 4 (see ‘Methods’). Definition for high diet quality: MDS ≥ 8.5 points; high PA: PAEE ≥ 5 kJ/kg/day. MDS, Mediterranean diet score; PAEE, physical activity energy expenditure; PA, physical activity; DQ, diet quality. Groups were formed according to reaching a high (H) or low (L) level of baseline PA, baseline DQ, repeated PA and repeated DQ as follows: G1 = LLLL, G2 = LLHL, G3 = LLLH, G4 = LLHH, G5 = HLLL, G6 = HLHL, G7 = HLLH, G8 = HLHH, G9 = LHLL, G10 = LHHL, G11 = LHLH, G12 = LHHH, G13 = HHLL, G14 = HHHL, G15 = HHLH and G16 = HHHH

Figure 2 shows the 16 exposure trajectories of the four dichotomised PA and diet categories at baseline and at the repeated assessment (G1 to G16). The most prevalent trajectories were participants that maintained the same levels of PA and diet from baseline over time. Compared to the reference group with sustained low PAEE and low MDS, those consistently active but with poor quality diet (G6) had 17% lower mortality, those consistently maintaining a good quality diet but consistently inactive over time (G11) had 13% lower mortality, whereas those who maintained both high PA and diet quality over time (G16) had 22% lower mortality.

Among participants who increased their PA over time but without changes to their baseline diet quality (G2, G12), the risk reduction for all-cause mortality was only significant for those individuals who also maintained a high-quality diet (26% lower). Among individuals who were active at baseline and then decreased their PA but without changing their baseline diet quality (G5, G15), only those who consistently adhered to a high-quality diet demonstrated a significantly lower mortality risk (17% lower in G15, the largest group where changes in either behaviour were observed).

Similarly, among participants who improved their diet quality but without changing their PA levels (G3, G8), only those who consistently engaged in high levels of physical activity showed a lower risk of all-cause mortality (25% lower in G8). Furthermore, for those who transitioned from a high-quality diet to a low-quality diet while maintaining their baseline PA (G9, G14), the group that maintained high PA throughout the study exhibited a reduction in mortality risk, though this did not reach statistical significance (G14, HR [95% CI]: 0.83 [0.68 to 1.02]).

In the analysis stratified by baseline exposure combination and using maintenance of that combination over time as the reference (Fig. 2B), declines in either PA or diet were generally associated with higher mortality risk and improvements associated with lower mortality. Those who increased both PA and diet quality (G4) had a 40% lower mortality risk, while those who declined in both their PA and diet quality levels (G13) had a 47% higher risk of mortality, compared to those with sustained behaviours over time.

In sensitivity analyses for trajectories based on three level exposures at baseline and at repeated assessments, separately for physical activity and diet (but adjusted for the other), the largest differences in mortality were between the low and medium exposure levels, with little or no additional difference between medium and high (Additional file 1: Fig. S4). For the joint activity-diet analysis of change, and considering the three categories of behaviour maintainers, decreasers and increasers, improvements in either or both exposures were associated with lower mortality risk.

### Subgroup analyses

**Fig. 3:**
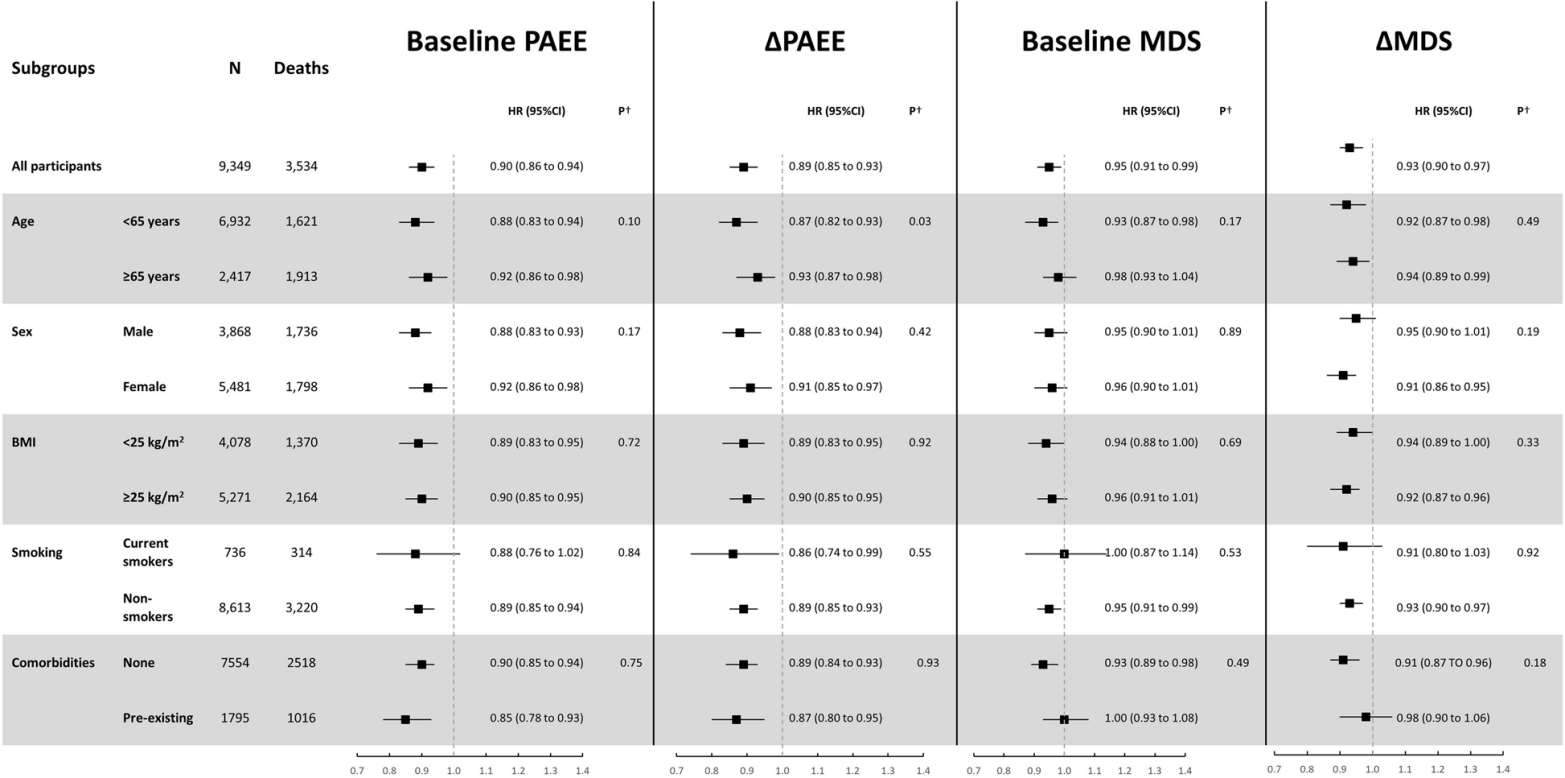
Association of mutually adjusted baseline and within-person changes in PAEE and MDS with all-cause mortality in different strata of baseline age, sex, BMI, smoking status and pre-existing comorbidities per 1-SD difference in each exposure, in the EPIC-Norfolk Study. Analysis is based on model 4 (see ‘Methods’). MDS, Mediterranean diet score; PAEE, physical activity energy expenditure. 1-SD increment in baseline PAEE equals to 4.64 kJ/kg/day, in ΔPAEE equals to 0.65 kJ/kg/day per year, in baseline MDS equals to 1.30 points and in ΔMDS equals to 0.33 points per year. Pre-existing comorbidities were defined as having any of the following: cancer, cardiovascular disease, diabetes or hypertension. †*P* values for interaction in subgroups

Mortality associations for the four exposures expressed as continuous variables generally persisted in stratified analyses of the most comprehensively adjusted model (i.e. model 4) by potential effect modifiers at baseline (age, sex, BMI, smoking status and comorbidities, Fig. 3). Tests for subgroup interaction were non-significant for all stratified analyses except for ∆PAEE and age.

### Population impact

**Fig. 4:**
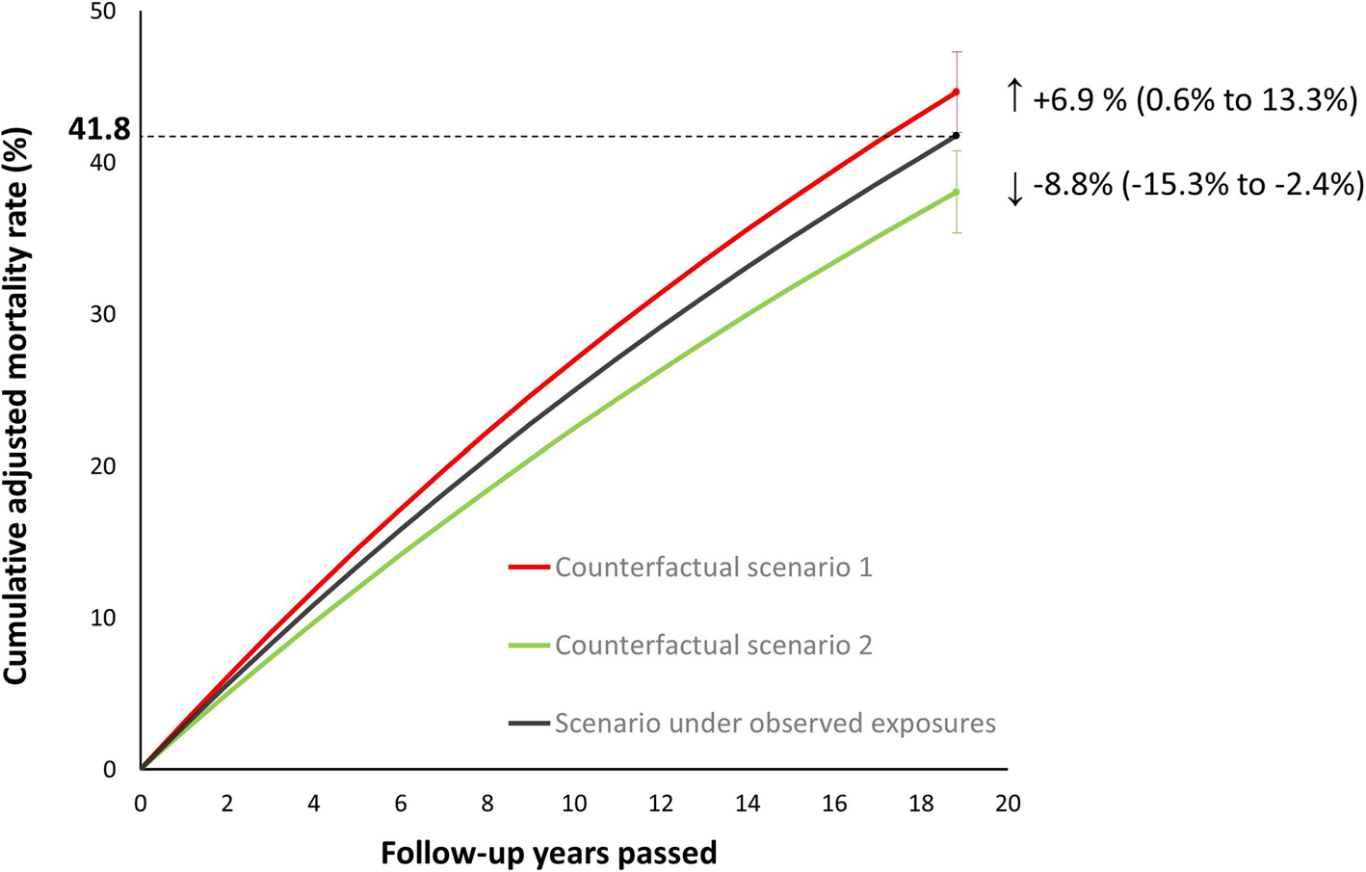
Population impact by estimating the cumulative adjusted mortality rate of the population during two decades of follow-up, under observed physical activity and diet exposures, compared with two counterfactual scenarios. Counterfactual scenario 1; applying the adjusted mortality rate of trajectory group 1 (G1, low PA and low diet quality at both assessments) to the whole population. Counterfactual scenario 2; applying the adjusted mortality rate of trajectory group 16 (G16, high PA and high diet quality at both assessments) to the whole population. See Fig. 2 and ‘Methods’ for the definition of each trajectory group. Error bars indicate 95% confidence intervals (95% CI) for percentage of changes in adjusted mortality rate of the population under counterfactual scenarios, compared with adjusted mortality rate under observed physical activity and diet exposures

Estimates of population impact were assessed under two different counterfactual scenarios (Fig. 4 and Additional file 1: Table S9). Using the adjusted mortality rate of the reference group (G1, low PA and low diet quality at both assessments) in the first counterfactual scenario indicated that compared to the adjusted results under observed PA and diet exposures, an additional 271 deaths (equivalent to 6.9% [95% CI: 0.6 to 13.3%] increase in the cumulative adjusted mortality rate of the population) would have been observed in the cohort during two decades of follow-up. These additional deaths did not happen within our cohort, meaning they were averted, as not all participants maintained consistently low levels of physical activity and diet quality throughout the assessment period. The greatest proportion of these averted deaths was from the group with consistently high PA and diet quality levels (G16, 32%). Other main contributors to potential deaths postponed were trajectory groups which had either a consistently high PA or high diet quality level (both accounting for about 15% each), or achieved a combined high PA and high diet quality level at the repeated assessment, despite one or both exposures being suboptimal at baseline (combined accounting for 28% of postponed deaths).

In the second counterfactual scenario, applying the adjusted mortality rate of those recording high PA and high diet quality at both measurements (G16) to all other trajectory groups would have led to 345 fewer deaths in the cohort than the adjusted deaths under observed PA and diet exposures, yielding the estimate that cumulative adjusted mortality rate of the population would have been decreased by 8.8% (95% CI: 2.4 to 15.3%) if all the participants followed this scenario.

## Discussion

The findings from this prospective study suggest that higher levels of PA and diet quality, at baseline and over time, separately and in combination, are associated with lower all-cause and cause-specific mortality. This suggests that both the starting point and how these health behaviours change throughout adulthood are relevant for mortality.

Examining specific longitudinal trajectories of PA and diet quality revealed that compared with being consistently physically inactive and eating a low-quality diet, maintaining either high PA or high diet quality over time was associated with around a 15% lower mortality risk, while maintaining both behaviours at a higher level over time was associated with greater benefit (22% lower mortality risk).

In subgroups based on baseline health behaviours, compared with the stable trajectory within each stratum, the biggest differences in mortality risk were observed in the groups who observed the greatest health behaviour changes: 40% lower risk for moving from combined low PA and diet quality to combined high levels of these behaviours and 47% higher risk for moving from combined high PA and diet quality to combined low levels of these behaviours. Furthermore, results from the stratified analyses based on baseline characteristics generally followed the main findings for all exposures. Despite the lack of significance for some exposures in specific subgroups, the magnitude and directions of associations were similar across subgroups.

Taken together, our findings, therefore, emphasise the importance of both activity and diet behaviours and argue that we should not assume that the negative consequences of a poor diet can be overcome with engagement in PA over the long term or vice versa. This argument is further supported by the absence of interaction between PA and diet quality, indicating that neither PA nor diet quality would obscure each other’s influence on longevity.

It is well established that regular PA and healthy diet are important modifiable health behaviours lowering the risk of premature mortality [25–27] and a wide range of public health guidelines promote messages to improve these health behaviours. A particular challenge has been around the notion that one may exercise routinely to offset the negative consequences of a ‘bad diet’. Support for this notion has come mainly from small-scale overfeeding studies with short duration of follow-ups [28, 29]. Using the highest vs. lowest tertiles of adherence to MED and PA, two Spanish cohort studies reported that joint adherence to higher levels of MED and PA was more beneficial for longevity than high adherence to each of them alone [9, 10]. Specifically, joint adherence to both behaviours was associated with ~ 65 to ~ 75% lower risk for all-cause mortality, while separately, MED alone and PA alone were associated with ~ 35% and ~ 50% lower risk for all-cause mortality, respectively. Nevertheless, a relevant aspect that remained unaddressed in these studies was whether one could ‘outrun’ a poor diet with increased PA or, conversely, ‘get away’ with being physically inactive by adhering to a healthy diet. In our study, however, we were able to explore these questions by adopting a comprehensive modelling approach, incorporating baseline and within-person changes in both PA and diet as mutual exposures, analysing the multiplicative and additive interactions between these factors and considering them as joint exposure trajectories.

More recently, findings from 346,627 participants of the UK Biobank showed that although high diet quality was not independently associated with all-cause and CVD mortality, in the joint analysis, the highest quartile of PA engagement combined with the category of highest diet quality score was associated with lower all-cause mortality, CVD mortality and cancer mortality [11]. However, a simplified dietary index was used in UK Biobank based on intake of three food items (fruit and vegetables; fish; and red and processed meat) which may not capture overall healthy diet quality as well as the Mediterranean diet score does. This may in part account for the lack of associations that were observed between diet and mortality outcomes in that study [11]. Overall, our findings are in line with the results reported by studies in Spanish and UK cohorts; however, our repeated assessment of PAEE and MDS allows an even stronger inference about the joint effect of diet and PA including the simultaneous within-person changes in these behaviours.

To the best of our knowledge, our study is the first to investigate the associations of changes in PA and diet quality with mortality outcomes considering within-person changes in the health behaviours simultaneously as mutual exposures. A recent systematic review has provided evidence on changes in PA using repeated measures of the PA exposure, showing that being consistently or increasingly active in adults is associated with lower all-cause and CVD mortality [30]. This association was consistent across a range of applied methodological approaches, with varied categorisation strategies for exposures, methods of analysis and covariates included in the models. In line with this, we report that one SD increase in PA over time was associated with 11% lower all-cause mortality and CVD mortality. Notably, this increase in PA is equivalent to a PAEE of 0.65 kJ/kg/day per year, which, for example, could be achieved by adding 8 min of brisk walking, over a period of 3 years. For cancer mortality, there is sparse evidence with inconsistent results, ranging from inverse association between baseline PA or increasing PA over time and cancer mortality [16, 31] to no association [32, 33]. Our current results support the previous findings in the EPIC-Norfolk population [16] and show a 9% lower risk of cancer mortality per one SD increase in PA over time.

Data from different US population-based cohort studies have shown that changes in diet quality over time are associated with lower all-cause and CVD mortality [34–38]. Similarly, we currently report a 7% lower all-cause mortality and 6% lower CVD mortality per one SD increase in MDS over time. This improvement in MDS can be accomplished by replacing one weekly serving of red meat with fish, for example, for an individual who usually consumes four servings of red meat per week, over a period of 3 years. We further observed a significant association between ΔMDS and cancer mortality, which is supported by recent systematic reviews and meta-analyses [1, 39] but is in contrast to findings from the Nurses’ Health Study, the Health Professionals Follow-up Study and the Multiethnic Cohort Study, examining changes in the Mediterranean diet score [34, 36]. It is noteworthy that studies based on these American cohorts did not consider concomitant changes in diet quality and PA as mutual exposures [34, 36], nor took into account the role of time-varying confounders [36], and hence did not examine whether following a Mediterranean-type diet may act as a marker of generally healthier decisions about other factors too, such as non-smoking or being physically active [12, 40].

### Strengths and limitations

Strengths of our research include a prospective study design with repeated assessments of both PA and diet and long subsequent follow-up in a large sample size, enabling temporality of associations to be examined while accounting for several confounding factors and including time-varying confounding factors. Linkage of all participants to national registries provided the opportunity to evaluate all-cause and cause-specific mortality. Our analyses were comprehensive including independent and joint assessment of exposures with explicit consideration of baseline values and changes in them, the development of different combined trajectories of PA and diet quality, quantification of population health impact, tests of interaction based on multiplicative and additive scales, stratified analysis and various sensitivity analyses.

Our study has some limitations. Since our research objective required participants with repeated assessments of both PA and diet, a large proportion (63.5%) of baseline participants of the EPIC-Norfolk Study were excluded, making our findings susceptible to healthy cohort bias, with the included sample having higher levels of baseline PAEE and MDS and lower levels of cardiometabolic risk factors compared with the excluded sample. Nonetheless, this issue does not undermine the internal validity of our study and we minimised biases by imputation of missing covariates. Furthermore, because in our healthier (analytical) sample, we still observed strong associations, we can speculate that these findings might have been even more pronounced in the whole population had the data been available. Therefore, if anything, our current results could be considered conservative. In addition, associations for baseline-only exposures to mortality in this sample are similar to previously published results from the full cohort [21, 41]. PA and diet were assessed by self-report and thus susceptible to recall bias and social desirability bias; however, both exposure measures have been successfully validated against objective criterion measures, and the impact of measurement error is likely to result in estimates of association which are conservative. The repeated assessments of PA and diet did not take place at the same time, and on average, more time had elapsed between PA measurements than between diet measurements, impacting the direct comparability between associations of change in these two health behaviours. However, our sensitivity analysis using HC3 as the end of the exposure assessment period yielded similar findings as from the primary analysis, suggesting the latent behavioural trajectories over time can be assessed with either approach when expressed as annual changes. Furthermore, we standardised our exposures to SD units which facilitated comparisons. Despite comprehensive adjustment for confounding in statistical models, residual confounding is possible from imprecisely measured or unmeasured factors, such as prevalent musculoskeletal diseases at the baseline. Our study is observational and cannot firmly establish causality of associations but conducting long-term large randomised controlled studies of health behaviours would be challenging. One potential limitation of our study is the possibility of multiple testing issue; however, we believe this concern is mitigated by our cautious and balanced interpretation of results, where we prioritised the magnitude of association and consistency of findings across different analyses over isolated significant results. Our findings are based on an English middle-aged to elderly population of European descent which limits generalisability to younger populations, different sociodemographic or ethnic characteristics, or populations living in other regions of the world.

### Public health implications

There has been an ongoing debate about whether a poor diet can be compensated for by engaging in high levels of physical activity or vice versa. Our findings contradict this viewpoint by showing the independent protective association of baseline and changes in both PA and diet against mortality in various mutually adjusted models. Furthermore, the lack of any significant interaction between the baseline and delta exposures highlights that possible changes in a person’s PA and diet quality that could potentially occur in the future may be as important as current status of these health behaviours.

Our estimate of population impact suggests that an additional 271 deaths would have been observed, had the whole population maintained low levels of PA and diet quality from baseline to follow-up (increasing the adjusted mortality rate by 6.9%, 95% CI: 0.6 to 13.3%). The prevention of these pre-mature deaths was achieved mainly by the relatively large group who were consistently active and consumed a high-quality diet (32% contribution). The two groups who were also stable for both exposures over time but where only one behaviour was optimal were each responsible for preventing a further 15% of these deaths. These findings imply that health benefits are achievable by optimisation of both PA and diet quality and if anything, they work in parallel rather than playing against each other. In the counterfactual ideal scenario, where the whole population would have maintained high levels of PA and diet quality from baseline to follow-up, 345 additional deaths could have been prevented (decreasing the adjusted mortality rate by 8.8%, 95% CI: 2.4 to 15.3%). Overall, while this cohort had observed a meaningful achievement of ~ 7% prevention of premature deaths through optimising PA and diet quality, there is still potential for a further ~ 9% improvement with greater sustained adherence to high PA and high diet quality levels.

## Conclusions

In summary, our findings suggest both being active and eating a good quality diet, as well as building up to these healthy habits over time, are associated with lower mortality risk. As these associations were all independent of each other, the public health message is that it is not too late to adopt an active lifestyle and improve diet quality in mid or late adulthood in order to live longer.

## Supplementary Information

Additional file 1: Fig. S1 Flow diagram illustrating selection of the analytical sample in the EPIC-Norfolk Study. Table S1 Comparison of baseline characteristics in the analytical sample according to all-cause mortality outcome at the end of follow-up in the EPIC-Norfolk Study. Table S2 Comparison of baseline characteristics in the analytical sample and those excluded from analysis in the EPIC-Norfolk population. Table S3 Associations of mutually adjusted exposures with mortality outcomes in the EPIC-Norfolk Study (complete case analysis). Table S4 Associations of mutually adjusted exposures with subtypes of CVD and cancer mortality outcomes in the EPIC-Norfolk Study. Table S5 Tests of interaction between different combinations of the exposures for all-cause mortality outcome in the EPIC-Norfolk Study. Table S6 Associations of mutually adjusted cumulative exposures with mortality outcomes in the EPIC-Norfolk Study. Fig. S2 EPIC-Norfolk Study design and timeline, considering health check 3 as the end of assessment period. Table S7 Associations of mutually adjusted exposures with mortality in the EPIC-Norfolk Study, considering health check 3 as the end of the assessment period. Table S8 Associations of mutually adjusted exposures with mortality in the EPIC-Norfolk Study, excluding deaths that occurred within 2 years of the last measurement. Fig. S3 Dose–response relationship between PA and diet exposures and all-cause mortality fitted by using a Cox proportional hazards with fractional polynomial in the EPIC-Norfolk Study. Fig. S4 Associations of different trajectories of PA and diet with all-cause mortality in the EPIC-Norfolk Study, based on three-by-three levels of exposures. Table S9 Population impact by estimating the differences in total number of deaths that could have been potentially observed under two different counterfactual scenarios.

## Data Availability

The data cannot be made openly available because of ethical and legal considerations. Non-identifiable data can be made available to bona-fide researchers on submission of a reasonable request to datasharing@mrc-epid.cam.ac.uk. The principles and processes for accessing and sharing data are outlined in the MRC Epidemiology Unit Data Access & Data Sharing Policy.

## Abbreviations

PA: Physical activity
MED: Mediterranean-type diet
EPIC-Norfolk: European Prospective Investigation into Cancer in Norfolk
PAEE: Physical activity energy expenditure
MDS: Mediterranean diet score
ΔPAEE: Within-person changes in physical activity energy expenditure
ΔMDS: Within-person changes in Mediterranean diet score
HR: Hazard ratio
CVD: Cardiovascular disease
HC: Health check
PFU: Postal follow-up
OPA: Occupational physical activity
LTPA: Leisure-time physical activity
FFQ: Food frequency questionnaire
BMI: Body mass index
SBP: Systolic blood pressure
DBP: Diastolic blood pressure
TG: Triglycerides
LDL-C: Low-density lipoprotein cholesterol
HDL-C: High-density lipoprotein cholesterol
GCE: General Certificate of Education
ICD: International classification of diseases
IHD: Ischemic heart disease
G[number]: Group [number]
DM: Diabetes mellitus
SD: Standard deviation

## References

[1] Morze J, Danielewicz A, Przybyłowicz K, Zeng H, Hoffmann G, Schwingshackl L. An updated systematic review and meta-analysis on adherence to Mediterranean diet and risk of cancer. Eur J Nutr. 2021;60(3):1561–86. 32770356 10.1007/s00394-020-02346-6 PMC7987633

[2] Rosato V, Temple NJ, La Vecchia C, Castellan G, Tavani A, Guercio V. Mediterranean diet and cardiovascular disease: a systematic review and meta-analysis of observational studies. Eur J Nutr. 2019;58(1):173–91. 29177567 10.1007/s00394-017-1582-0

[3] Becerra-Tomás N, Blanco Mejía S, Viguiliouk E, Khan T, Kendall CWC, Kahleova H, et al. Mediterranean diet, cardiovascular disease and mortality in diabetes: a systematic review and meta-analysis of prospective cohort studies and randomized clinical trials. Crit Rev Food Sci Nutr. 2020;60(7):1207–27. 30676058 10.1080/10408398.2019.1565281

[4] Eleftheriou D, Benetou V, Trichopoulou A, La Vecchia C, Bamia C. Mediterranean diet and its components in relation to all-cause mortality: meta-analysis. Br J Nutr. 2018;120(10):1081–97. 30401007 10.1017/S0007114518002593

[5] Garcia L, Pearce M, Abbas A, Mok A, Strain T, Ali S, et al. Non-occupational physical activity and risk of cardiovascular disease, cancer and mortality outcomes: a dose-response meta-analysis of large prospective studies. Br J Sports Med. 2023. 10.1136/bjsports-2022-105669 PMC10423495 36854652

[6] Posadzki P, Pieper D, Bajpai R, Makaruk H, Könsgen N, Neuhaus AL, et al. Exercise/physical activity and health outcomes: an overview of Cochrane systematic reviews. BMC Public Health. 2020;20(1):1724. 33198717 10.1186/s12889-020-09855-3 PMC7670795

[7] Caprara G. Mediterranean-type dietary pattern and physical activity: the winning combination to counteract the rising burden of non-communicable diseases (NCDs). Nutrients. 2021;13(2). 10.3390/nu13020429 PMC7910909 33525638

[8] Baranowski T. Why combine diet and physical activity in the same international research society? Int J Behav Nutr Phys Act. 2004;1(1):2. 15171787 10.1186/1479-5868-1-2 PMC416564

[9] Cárdenas-Fuentes G, Subirana I, Martinez-Gonzalez MA, Salas-Salvadó J, Corella D, Estruch R, et al. Multiple approaches to associations of physical activity and adherence to the Mediterranean diet with all-cause mortality in older adults: the PREvención con DIeta MEDiterránea study. Eur J Nutr. 2019;58(4):1569–78. 29696401 10.1007/s00394-018-1689-y

[10] Alvarez-Alvarez I, Zazpe I, Pérez de Rojas J, Bes-Rastrollo M, Ruiz-Canela M, Fernandez-Montero A, et al. Mediterranean diet, physical activity and their combined effect on all-cause mortality: the Seguimiento Universidad de Navarra (SUN) cohort. Prev Med. 2018;106:45–52. 10.1016/j.ypmed.2017.09.021 28964855

[11] Ding D, Van Buskirk J, Nguyen B, Stamatakis E, Elbarbary M, Veronese N, et al. Physical activity, diet quality and all-cause cardiovascular disease and cancer mortality: a prospective study of 346 627 UK Biobank participants. Br J Sports Med. 2022. 10.1136/bjsports-2021-105195 35811091

[12] Williamson EJ, Polak J, Simpson JA, Giles GG, English DR, Hodge A, et al. Sustained adherence to a Mediterranean diet and physical activity on all-cause mortality in the Melbourne Collaborative Cohort Study: application of the g-formula. BMC Public Health. 2019;19(1):1733. 31878916 10.1186/s12889-019-7919-2 PMC6933918

[13] Day N, Oakes S, Luben R, Khaw KT, Bingham S, Welch A, et al. EPIC-Norfolk: study design and characteristics of the cohort. European Prospective Investigation of Cancer. Br J Cancer. 1999;80 Suppl 1:95–103. 10466767

[14] Peters T, Brage S, Westgate K, Franks PW, Gradmark A, Tormo Diaz MJ, et al. Validity of a short questionnaire to assess physical activity in 10 European countries. Eur J Epidemiol. 2012;27(1):15–25. 22089423 10.1007/s10654-011-9625-y PMC3292724

[15] Wareham NJ, Jakes RW, Rennie KL, Schuit J, Mitchell J, Hennings S, et al. Validity and repeatability of a simple index derived from the short physical activity questionnaire used in the European Prospective Investigation into Cancer and Nutrition (EPIC) study. Public Health Nutr. 2003;6(4):407–13. 12795830 10.1079/PHN2002439

[16] Mok A, Khaw KT, Luben R, Wareham N, Brage S. Physical activity trajectories and mortality: population based cohort study. BMJ. 2019;365: l2323. 31243014 10.1136/bmj.l2323 PMC6592407

[17] Bingham SA, Gill C, Welch A, Day K, Cassidy A, Khaw KT, et al. Comparison of dietary assessment methods in nutritional epidemiology: weighed records v. 24 h recalls, food-frequency questionnaires and estimated-diet records. Br J Nutr. 1994;72(4):619–43. 10.1079/bjn19940064 7986792

[18] Bingham SA, Gill C, Welch A, Cassidy A, Runswick SA, Oakes S, et al. Validation of dietary assessment methods in the UK arm of EPIC using weighed records, and 24-hour urinary nitrogen and potassium and serum vitamin C and carotenoids as biomarkers. Int J Epidemiol. 1997;26(Suppl 1):S137–51. 9126542 10.1093/ije/26.suppl_1.s137

[19] Bingham SA, Cassidy A, Cole TJ, Welch A, Runswick SA, Black AE, et al. Validation of weighed records and other methods of dietary assessment using the 24 h urine nitrogen technique and other biological markers. Br J Nutr. 1995;73(4):531–50. 7794870 10.1079/bjn19950057

[20] Bach-Faig A, Berry EM, Lairon D, Reguant J, Trichopoulou A, Dernini S, et al. Mediterranean diet pyramid today. Science and cultural updates. Public Health Nutr. 2011;14(12a):2274–84. 10.1017/S1368980011002515 22166184

[21] Tong TY, Wareham NJ, Khaw KT, Imamura F, Forouhi NG. Prospective association of the Mediterranean diet with cardiovascular disease incidence and mortality and its population impact in a non-Mediterranean population: the EPIC-Norfolk study. BMC Med. 2016;14(1):135. 27679997 10.1186/s12916-016-0677-4 PMC5041408

[22] Hayat SA, Luben R, Keevil VL, Moore S, Dalzell N, Bhaniani A, et al. Cohort profile: a prospective cohort study of objective physical and cognitive capability and visual health in an ageing population of men and women in Norfolk (EPIC-Norfolk 3). Int J Epidemiol. 2014;43(4):1063–72. 23771720 10.1093/ije/dyt086 PMC4121549

[23] White IR, Royston P, Wood AM. Multiple imputation using chained equations: issues and guidance for practice. Stat Med. 2011;30(4):377–99. 21225900 10.1002/sim.4067

[24] Andersson T, Alfredsson L, Källberg H, Zdravkovic S, Ahlbom A. Calculating measures of biological interaction. Eur J Epidemiol. 2005;20(7):575–9. 16119429 10.1007/s10654-005-7835-x

[25] Global burden of 87 risk factors in 204 countries and territories, 1990–2019: a systematic analysis for the Global Burden of Disease Study 2019. Lancet. 2020;396(10258):1223–49. 10.1016/S0140-6736(20)30752-2 PMC7566194 33069327

[26] O’Connor EA, Evans CV, Rushkin MC, Redmond N, Lin JS. Behavioral counseling to promote a healthy diet and physical activity for cardiovascular disease prevention in adults with cardiovascular risk factors: updated evidence report and systematic review for the US Preventive Services Task Force. JAMA. 2020;324(20):2076–94. 33231669 10.1001/jama.2020.17108 PMC13556284

[27] Clinton SK, Giovannucci EL, Hursting SD. The World Cancer Research Fund/American Institute for Cancer Research third expert report on diet, nutrition, physical activity, and cancer: impact and future directions. J Nutr. 2020;150(4):663–71. 31758189 10.1093/jn/nxz268 PMC7317613

[28] Walhin JP, Richardson JD, Betts JA, Thompson D. Exercise counteracts the effects of short-term overfeeding and reduced physical activity independent of energy imbalance in healthy young men. J Physiol. 2013;591(24):6231–43. 24167223 10.1113/jphysiol.2013.262709 PMC3892474

[29] Duval C, Rouillier MA, Rabasa-Lhoret R, Karelis AD. High intensity exercise: can it protect you from a fast food diet? Nutrients. 2017;9(9). 10.3390/nu9090943 PMC5622703 28846611

[30] Yang Y, Dixon-Suen SC, Dugué PA, Hodge AM, Lynch BM, English DR. Physical activity and sedentary behaviour over adulthood in relation to all-cause and cause-specific mortality: a systematic review of analytic strategies and study findings. Int J Epidemiol. 2022;51(2):641–67. 34480556 10.1093/ije/dyab181

[31] Saint-Maurice PF, Coughlan D, Kelly SP, Keadle SK, Cook MB, Carlson SA, et al. Association of leisure-time physical activity across the adult life course with all-cause and cause-specific mortality. JAMA Netw Open. 2019;2(3): e190355. 30848809 10.1001/jamanetworkopen.2019.0355 PMC6484624

[32] Wolin KY, Patel AV, Campbell PT, Jacobs EJ, McCullough ML, Colditz GA, et al. Change in physical activity and colon cancer incidence and mortality. Cancer Epidemiol Biomarkers Prev. 2010;19(12):3000–4. 20978171 10.1158/1055-9965.EPI-10-0764 PMC3139493

[33] Gregg EW, Cauley JA, Stone K, Thompson TJ, Bauer DC, Cummings SR, et al. Relationship of changes in physical activity and mortality among older women. JAMA. 2003;289(18):2379–86. 12746361 10.1001/jama.289.18.2379

[34] Sotos-Prieto M, Bhupathiraju SN, Mattei J, Fung TT, Li Y, Pan A, et al. Association of changes in diet quality with total and cause-specific mortality. N Engl J Med. 2017;377(2):143–53. 28700845 10.1056/NEJMoa1613502 PMC5589446

[35] Baden MY, Liu G, Satija A, Li Y, Sun Q, Fung TT, et al. Changes in plant-based diet quality and total and cause-specific mortality. Circulation. 2019;140(12):979–91. 31401846 10.1161/CIRCULATIONAHA.119.041014 PMC6746589

[36] Park SY, Kang M, Shvetsov YB, Setiawan VW, Boushey CJ, Haiman CA, et al. Diet quality and all-cause and cancer-specific mortality in cancer survivors and non-cancer individuals: the Multiethnic Cohort Study. Eur J Nutr. 2022;61(2):925–33. 34657186 10.1007/s00394-021-02700-2 PMC8857026

[37] Kang M, Boushey CJ, Shvetsov YB, Setiawan VW, Paik HY, Wilkens LR, et al. Changes in diet quality over 10 years and subsequent mortality from cardiovascular disease in the Multiethnic Cohort Study. Nutrients. 2023;15(15). 10.3390/nu15153482 PMC10421371 37571419

[38] Sullivan VK, Appel LJ, Anderson CAM, Tan TC, Brown J, Ricardo AC, et al. Changes in diet quality, risk of CKD progression, and all-cause mortality in the CRIC Study. Am J Kidney Dis. 2023;81(5):621–4. 36455682 10.1053/j.ajkd.2022.09.020 PMC10228419

[39] Schwingshackl L, Schwedhelm C, Galbete C, Hoffmann G. Adherence to Mediterranean diet and risk of cancer: an updated systematic review and meta-analysis. Nutrients. 2017;9(10). 10.3390/nu9101063 PMC5691680 28954418

[40] Knoops KT, de Groot LC, Kromhout D, Perrin AE, Moreiras-Varela O, Menotti A, et al. Mediterranean diet, lifestyle factors, and 10-year mortality in elderly European men and women: the HALE project. JAMA. 2004;292(12):1433–9. 15383513 10.1001/jama.292.12.1433

[41] Khaw KT, Wareham N, Bingham S, Welch A, Luben R, Day N. Combined impact of health behaviours and mortality in men and women: the EPIC-Norfolk prospective population study. PLoS Med. 2008;5(1): e12. 18184033 10.1371/journal.pmed.0050012 PMC2174962

