# Supplementary material for: Combined associations of physical activity, diet quality and their changes over time with mortality: findings from the EPIC-Norfolk study, United Kingdom
Source: BMC Med. 2024 Oct 14;22:464. doi: 10.1186/s12916-024-03668-6 (PMC11476187; doi:10.1186/s12916-024-03668-6)
Supplement: Supplementary file 1 — Additional file 1: Fig. S1 Flow diagram illustrating selection of the analytical sample in the EPIC-Norfolk Study. Table S1 Comparison of baseline characteristics in the analytical sample according to all-cause mortality outcome at the end of follow-up in the EPIC-Norfolk Study. Table S2 Comparison of baseline characteristics in the analytical sample and those excluded from analysis in the EPIC-Norfolk population. Table S3 Associations of mutually adjusted exposures with mortality outcomes in the EPIC-Norfolk Study (complete case analysis). Table S4 Associations of mutually adjusted exposures with subtypes of CVD and cancer mortality outcomes in the EPIC-Norfolk Study. Table S5 Tests of interaction between different combinations of the exposures for all-cause mortality outcome in the EPIC-Norfolk Study. Table S6 Associations of mutually adjusted cumulative exposures with mortality outcomes in the EPIC-Norfolk Study. Fig. S2 EPIC-Norfolk Study design and timeline, considering health check 3 as the end of assessment period. Table S7 Associations of mutually adjusted exposures with mortality in the EPIC-Norfolk Study, considering health check 3 as the end of the assessment period. Table S8 Associations of mutually adjusted exposures with mortality in the EPIC-Norfolk Study, excluding deaths that occurred within 2 years of the last measurement. Fig. S3 Dose–response relationship between PA and diet exposures and all-cause mortality fitted by using a Cox proportional hazards with fractional polynomial in the EPIC-Norfolk Study. Fig. S4 Associations of different trajectories of PA and diet with all-cause mortality in the EPIC-Norfolk Study, based on three-by-three levels of exposures. Table S9 Population impact by estimating the differences in total number of deaths that could have been potentially observed under two different counterfactual scenarios. [file 12916_2024_3668_MOESM1_ESM.pdf]

Supporting information

Additional file 1:

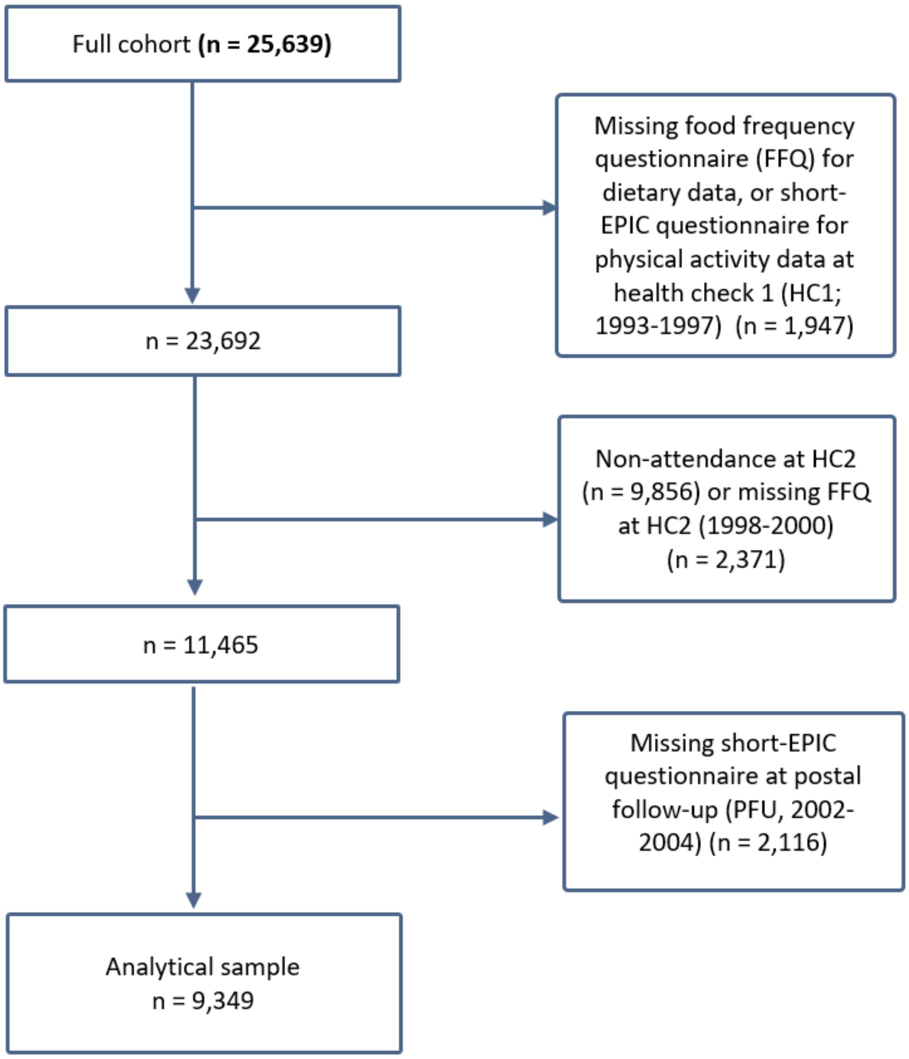

Figure S1 - Flow diagram illustrating selection of the analytical sample in the EPIC-Norfolk Study.

Table S1 - Comparison of baseline characteristics in the analytical sample according to all-cause mortality outcome at the end of follow-up in the EPIC-Norfolk Study

Characteristic	Alive (n=5,815)	Dead (n=3,534)	P-value
**Demographics**			
Age (years)	54.4 (7.0)	64.8 (7.1)	<0.001
Women (%)	63.3	50.9	<0.001
Education: GCSE/O level equivalent or below (%)	38.8	46.6	<0.001
Occupation: Unemployed to semi-skilled work (%)	14.6	17.1	0.001
Occupation: Skilled workers (%)	37.0	36.7	0.821
Occupation: Managers & professionals (%)	48.4	46.2	0.034
**Metabolic risk factors**			
Body mass index (kg/m^2)	25.6 (3.6)	26.3 (3.8)	<0.001
Systolic blood pressure (mmHg)	131.0 (16.4)	140.3 (18.2)	<0.001
Diastolic blood pressure (mmHg)	81.1 (10.5)	84.5 (11.2)	<0.001
Triglycerides (mmol/L)	1.65 (1.03)	1.88 (1.07)	<0.001
Total Cholesterol (mmol/L)	6.03 (1.12)	6.34 (1.16)	<0.001
HDL-Cholesterol (mmol/L)	1.46 (0.44)	1.39 (0.43)	<0.001
LDL-Cholesterol (mmol/L)	3.85 (1.01)	4.11 (1.03)	<0.001
**Self-reported comorbidities, medication, and family history**			
Diabetes mellitus (%)	0.7	2.7	<0.001
Cardiovascular diseases (%)	0	0.1	0.026
Cancer (%)	4.0	7.1	<0.001
Statins (%)	0.6	1.2	0.002
Anti-hypertensive drugs (%)	9.2	21.7	<0.001
Family history of Diabetes Mellitus (%)	14.0	12.9	0.128
Family history of myocardial infarction (%)	35.8	39.5	<0.001
**Health behaviours**			
MDS points	8.63 (1.31)	8.42 (1.27)	<0.001
PAEE (kJ/kg/day)	6.49 (4.58)	5.31 (4.63)	<0.001
Current smoker (%)	7.3	8.9	0.005
Former smoker (%)	35.7	46.0	<0.001
Energy intake (kcal/day)	2034.5 (582.8)	2100.6 (581.4)	<0.001
Alcohol (g/d)	8.4 (11.7)	8.7 (13.4)	0.21

HDL=high density lipoprotein; LDL=low density lipoprotein; MDS=Mediterranean diet score; PAEE=physical activity energy expenditure.

Table S2 - Comparison of baseline characteristics in the analytical sample and those excluded from analysis in the EPIC-Norfolk population.

**Characteristic**	**Analytical sample (n= 9,349)**	**Excluded (n=16,287)**	**P-value**
**Demographics**			
Age (years)	58.3 (8.7)	59.7 (9.6)	<0.001
Women (%)	58.6	52.5	<0.001
Education: GCSE/O level equivalent or below (%)	41.7	50.2	<0.001
Occupation: Unemployed to semi-skilled work (%)	15.5	20.6	
Skilled workers (%)	36.9	39.7	<0.001
Managers & Professionals (%)	47.5	39.7	
**Metabolic risk Factors**			
Body mass index (kg/m2)	25.9 (3.7)	26.6 (4.0)	<0.001
Systolic blood pressure (mmHg)	134.5 (17.7)	136.5 (18.9)	<0.001
Diastolic blood pressure (mmHg)	82.2 (10.9)	83.1 (11.5)	<0.001
Triglycerides (mmol/L)	1.73 (1.05)	1.9 (1.32)	<0.001
Total Cholesterol (mmol/L)	6.15 (1.15)	6.20 (1.18)	<0.001
HDL-Cholesterol (mmol/L)	1.43 (0.43)	1.40 (0.43)	<0.001
LDL-Cholesterol (mmol/L)	3.95 (1.03)	3.99 (1.04)	<0.001
Prevalent diseases, medication, and family history			
Diabetes mellitus (%)	1.4	2.8	<0.001
Cardiovascular diseases (%)	0.1	7.0	<0.001
Cancer (%)	5.1	5.7	0.055
Statins (%)	0.8	1.1	0.042
Anti-hypertensive drugs (%)	13.9	21.6	<0.001
Family history of diabetes mellitus (%)	13.6	12.4	0.004
Family history of myocardial infarction (%)	37.2	35.9	0.038
**Health behaviours**			
PAEE (kJ/kg/day)	6.04 (4.64)	5.13 (4.64)	<0.001
MDS score	8.55 (1.30)	8.36 (1.32)	<0.001
Current smoker (%)	7.9	13.8	<0.001
Former smoker (%)	39.6	43.3	<0.001
Energy intake (kcal/day)	2059 (583)	2036 (610)	0.005
Alcohol (g/d)	8.5 (12.4)	8.7 (13.3)	0.378

HDL=high density lipoprotein; LDL=low density lipoprotein; PAEE=physical activity energy expenditure; MDS=Mediterranean diet score.
Data are presented as mean (SD) or %.

Table S3 - Associations of mutually adjusted exposures with mortality outcomes in the EPIC-Norfolk Study (complete case analysis)†

	Exposures††	Model 1 HR (95% CI)	Model 2 HR (95% CI)	Model 3 HR (95% CI)	Model 4 HR (95% CI)
No. of participants		9,349	9,276	8,483	7,461
Time at risk		149,681	148,609	136,664	120,336
**All-cause mortality**					
No. of death		3,534	3,496	3,105	2,729
	Baseline PAEE	0.87 (0.83 to 0.91)***	0.88 (0.84 to 0.92)***	0.90 (0.86 to 0.94)***	0.90 (0.85 to 0.94)***
	ΔPAEE	0.87 (0.83 to 0.91)***	0.88 (0.84 to 0.91)***	0.90 (0.86 to 0.94)***	0.89 (0.85 to 0.93)***
	Baseline MDS	0.92 (0.88 to 0.95)***	0.94 (0.90 to 0.97)**	0.93 (0.89 to 0.97)**	0.94 (0.90 to 0.99)**
	ΔMDS	0.92 (0.88 to 0.95)***	0.93 (0.90 to 0.97)***	0.92 (0.89 to 0.96)***	0.91 (0.87 to 0.95)***
**CVD mortality**					
No. of death		2,978	2,949	2,599	2,283
	Baseline PAEE	0.87 (0.83 to 0.91)***	0.88 (0.84 to 0.92)***	0.91 (0.87 to 0.96)***	0.91 (0.86 to 0.96)***
	ΔPAEE	0.87 (0.83 to 0.92)***	0.88 (0.84 to 0.92)***	0.91 (0.86 to 0.96)***	0.91 (0.86 to 0.96)***
	Baseline MDS	0.91 (0.87 to 0.95)***	0.93 (0.89 to 0.97)**	0.91 (0.87 to 0.96)***	0.93 (0.88 to 0.97)**
	ΔMDS	0.92 (0.88 to 0.96)***	0.93 (0.89 to 0.97)**	0.92 (0.88 to 0.97)**	0.91 (0.87 to 0.96)***
**Cancer mortality**					
No. of death		1,794	1,771	1,591	1,407
	Baseline PAEE	0.89 (0.84 to 0.95)***	0.90 (0.85 to 0.96)**	0.92 (0.86 to 0.98)**	0.91 (0.85 to 0.97)**
	ΔPAEE	0.88 (0.83 to 0.93)***	0.89 (0.84 to 0.94)***	0.90 (0.85 to 0.96)**	0.90 (0.84 to 0.96)**
	Baseline MDS	0.95 (0.90 to 1.00)	0.97 (0.92 to 1.02)	0.98 (0.92 to 1.04)	0.98 (0.92 to 1.05)
	ΔMDS	0.93 (0.88 to 0.98)**	0.94 (0.89 to 0.99)*	0.94 (0.89 to 1.00)*	0.92 (0.86 to 0.98)**

HR=Hazard ratio per SD difference in each exposure; CI=Confidence interval.

Covariates in Models:

Model 1: sex, age.

Model 2: variables in Model 1 + education, employment, and time updated variables for smoking.

Model 3: variables in Model 2 + family history of myocardial infarction, family history of diabetes mellitus, time updated variables for prevalent diabetes mellitus, prevalent cancer, prevalent cardiovascular diseases, and anti-hypertensive medication.

Model 4: variables in Model 3 + time updated variables for BMI, SBP, DBP, TG, LDL, HDL, and total energy intake.

† Level of statistical significance: p-value ≥ 0.05, unless indicated by asterisk.: * P-value < 0.05; ** P-value < 0.01; *** P-value < 0.001

†† SD increment in baseline PAEE equals to 4.64 kJ/kg/day, in ΔPAEE equals to 0.65 kJ/kg/day per year, in baseline MDS equals to 1.30 points, and in ΔMDS equals to 0.33 points per year

Number of participants with missing covariates: Smoking (at baseline): n=73, Smoking (at repeated assessment): n=71, Education level: n=4, Self-reported comorbidities: n=801, Family history of MI: n=5, BMI (at baseline): n=5, BMI (at repeated assessment): n=17, SBP (at baseline): n=18, SBP (at repeated assessment): n=16, DBP (at baseline): n=16, DBP (at repeated assessment): n=16, TG (at baseline): n=489, TG (at repeated assessment): n=489, LDL (at baseline): n=722, LDL (at repeated assessment): n=498, HDL (at baseline): n=722, HDL (at repeated assessment): n=496, Energy intake (at baseline): n=19, Energy intake (at repeated assessment): n=38

Table S4 - Associations of mutually adjusted exposures with subtypes of CVD and cancer mortality outcomes in the EPIC-Norfolk Study†

Outcome	Exposures††	Model 1 HR (95% CI)	Model 2 HR (95% CI)	Model 3 HR (95% CI)	Model 4 HR (95% CI)
**IHD mortality (1,240 deaths)**	Baseline PAEE	0.82 (0.76 to 0.88)***	0.83 (0.77 to 0.89)***	0.88 (0.82 to 0.95)**	0.89 (0.83 to 0.96)**
	ΔPAEE	0.85 (0.78 to 0.91)***	0.86 (0.79 to 0.92)***	0.88 (0.81 to 0.95)**	0.88 (0.82 to 0.95)**
	Baseline MDS	0.92 (0.87 to 0.98)*	0.95 (0.89 to 1.01)	0.94 (0.88 to 1.00)	0.95 (0.89 to 1.02)
	ΔMDS	0.90 (0.84 to 0.96)**	0.91 (0.86 to 0.97)**	0.92 (0.86 to 0.98)*	0.91 (0.85 to 0.97)**
**Stroke mortality (976 deaths)**	Baseline PAEE	0.88 (0.81 to 0.96)**	0.89 (0.82 to 0.97)**	0.92 (0.85 to 1.00)*	0.91 (0.83 to 0.98)*
	ΔPAEE	0.89 (0.82 to 0.97)**	0.90 (0.83 to 0.98)*	0.91 (0.84 to 0.99)*	0.90 (0.83 to 0.98)*
	Baseline MDS	0.87 (0.81 to 0.94)***	0.88 (0.82 to 0.95)**	0.88 (0.82 to 0.95)**	0.90 (0.84 to 0.98)*
	ΔMDS	0.87 (0.80 to 0.93)***	0.87 (0.81 to 0.94)***	0.87 (0.81 to 0.94)***	0.87 (0.81 to 0.94)**
**Lung cancer mortality (169 deaths)**	Baseline PAEE	0.68 (0.55 to 0.83)***	0.72 (0.58 to 0.88)**	0.73 (0.59 to 0.90)**	0.74 (0.60 to 0.90)**
	ΔPAEE	0.79 (0.64 to 0.96)*	0.82 (0.67 to 1.00)	0.84 (0.68 to 1.03)	0.84 (0.68 to 1.03)
	Baseline MDS	0.87 (0.73 to 1.03)	0.99 (0.83 to 1.18)	0.97 (0.81 to 1.16)	1.00 (0.83 to 1.20)
	ΔMDS	0.87 (0.73 to 1.03)	0.94 (0.79 to 1.11)	0.92 (0.79 to 1.10)	0.95 (0.79 to 1.13)
**Prostate cancer mortality (293 deaths)**	Baseline PAEE	0.93 (0.81 to 1.07)	0.93 (0.81 to 1.07)	0.95 (0.83 to 1.10)	0.92 (0.80 to 1.07)
	ΔPAEE	1.03 (0.90 to 1.19)	1.04 (0.91 to 1.19)	1.08 (0.94 to 1.23)	1.06 (0.92 to 1.22)
	Baseline MDS	0.97 (0.88 to 1.11)	0.98 (0.85 to 1.11)	0.99 (0.87 to 1.13)	1.01 (0.88 to 1.16)
	ΔMDS	0.99 (0.87 to 1.14)	0.99 (0.87 to 1.13)	1.00 (0.88 to 1.15)	1.02 (0.89 to 1.17)

Continue in next page

Table S4 (continued)

Outcome	Exposures††	Model 1 HR (95% CI)	Model 2 HR (95% CI)	Model 3 HR (95% CI)	Model 4 HR (95% CI)
**Breast cancer mortality (184 deaths)**	Baseline PAEE	0.90 (0.74 to 1.09)	0.90 (0.74 to 1.10)	0.93 (0.76 to 1.13)	0.90 (0.74 to 1.11)
	ΔPAEE	0.96 (0.79 to 1.16)	0.96 (0.79 to 1.16)	1.00 (0.83 to 1.02)	1.00 (0.83 to 1.21)
	Baseline MDS	0.88 (0.75 to 1.05)	0.89 (0.75 to 1.06)	0.86 (0.72 to 1.03)	0.88 (0.74 to 1.06)
	ΔMDS	0.87 (0.74 to 1.02)	0.87 (0.74 to 1.03)	0.84 (0.71 to 1.00)*	0.83 (0.70 to 0.98)*
**GI cancer mortality (352 deaths)**	Baseline PAEE	0.90 (0.79 to 1.03)	0.91 (0.80 to 1.04)	0.93 (0.82 to 1.07)	0.94 (0.82 to 1.08)
	ΔPAEE	0.87 (0.76 to 1.00)*	0.88 (0.77 to 1.00)	0.91 (0.80 to 1.05)	0.92 (0.80 to 1.06)
	Baseline MDS	0.91 (0.81 to 1.03)	0.93 (0.82 to 1.05)	0.93 (0.82 to 1.05)	0.91 (0.89 to 1.04)
	ΔMDS	0.93 (0.82 to 1.05)	0.94 (0.83 to 1.06)	0.93 (0.82 to 1.05)	0.90 (0.80 to 1.02)

HR=Hazard ratio per SD difference in each exposure; CI=Confidence interval.
Covariates in Models:

Model 1: sex, age (only age for prostate and breast cancer mortality).

Model 2: variables in Model 1 + education, employment, and time updated variables for smoking.

Model 3: variables in Model 2 + family history of myocardial infarction, family history of diabetes mellitus, time updated variables for prevalent diabetes mellitus, prevalent cancer, prevalent cardiovascular diseases, and anti-hypertensive medication.

Model 4: variables in Model 3 + time updated variables for BMI, SBP, DBP, TG, LDL, HDL, and total energy intake.

† Level of statistical significance: p-value ≥ 0.05, unless indicated by asterisk.

* P-value < 0.05

** P-value < 0.01

*** P-value < 0.001

†† SD increment in baseline PAEE equals to 4.64 kJ/kg/day, in ΔPAEE equals to 0.65 kJ/kg/day per year, in baseline MDS equals to 1.30 points, and in ΔMDS equals to 0.33 points per year

Table S5 - Tests of interaction between different combinations of the exposures for all-cause mortality outcome in the EPIC-Norfolk Study†

Interaction terms in the model	Multiplicative interaction††	Additive interaction††		
	HR (95% CI)	RERI (95% CI)	AP (95% CI)	SI (95% CI)
a. Baseline PAEE × ΔPAEE	0.97 (0.94 to 1.00)	-0.045 (-0.207, 0.116)	-0.055 (-0.247, 0.137)	1.358 (0.336, 5.484)
b. Baseline MDS × ΔMDS	0.99 (0.96 to 1.02)	0.043 (-0.080, 0.167)	0.052 (-0.097, 0.202)	0.797 (0.445, 1.427)
c. Baseline PAEE × Baseline MDS	1.00 (0.97 to 1.04)	-0.007 (-0.134, 0.120)	-0.008 (-0.147, 0.132)	1.084 (0.236, 4.981)
d. ΔPAEE× ΔMDS	1.02 (0.99 to 1.06)	0.048 (-0.074, 0.171)	0.055 (-0.086, 0.197)	0.726 (0.364, 1.447)
	Likelihood-ratio test (p-value)††			
a + b	0.144			
c + d	0.420			
a + b + c + d	0.248			

HR, Hazard ratio; CI, Confidence interval RERI, the relative excess risk due to interaction; AP, the attributable proportion due to interaction; SI, the synergy index.

†Covariates in Model: sex, age, education, employment, family history of myocardial infarction, family history of diabetes mellitus, and time updated variables for smoking, prevalent diabetes mellitus, prevalent cancer, prevalent cardiovascular diseases, anti-hypertensive medication, BMI, SBP, DBP, TG, LDL, HDL, and total energy intake.

††Multiplicative interaction was tested by adding an interaction term between two exposures at a time and reporting the HR for that term from the regression model. Given all exposures are inversely related to mortality, an interaction HR of less than 1 indicates an amplification of the main effects. Additive interaction was assessed using the method previously described by Andersson T et al. (https://doi.org/10.1007/s10654-005-7835-x). When more than one interaction term was included in the model, a likelihood ratio test was used to compare the model with multiple interaction terms to a model without any interaction terms.

Table S6 - Associations of mutually adjusted cumulative exposures with mortality outcomes in the EPIC-Norfolk Study†

Outcome	Exposures††	Model 1<br>HR (95% CI)	Model 2<br>HR (95% CI)	Model 3<br>HR (95% CI)	Model 4<br>HR (95% CI)
**All-cause mortality (3534 deaths)**	Cumulative PAEE	0.89 (0.86 to 0.92)***	0.90 (0.87 to 0.93)***	0.92 (0.88 to 0.95)***	0.91 (0.88 to 0.95)***
	Cumulative MDS	0.92 (0.89 to 0.95)***	0.94 (0.91 to 0.97)**	0.94 (0.91 to 0.97)***	0.95 (0.92 to 0.99)*
**CVD mortality (2,978 deaths)**	Cumulative PAEE	0.89 (0.85 to 0.93)***	0.90 (0.86 to 0.94)***	0.93 (0.88 to 0.96)***	0.92 (0.89 to 0.96)***
	Cumulative MDS	0.91 (0.88 to 0.95)***	0.93 (0.90 to 0.97)***	0.93 (0.89 to 0.97)***	0.95 (0.91 to 0.98)**
**Cancer mortality (1,794 deaths)**	Cumulative PAEE	0.91 (0.87 to 0.96)***	0.92 (0.87 to 0.97)**	0.94 (0.89 to 0.99)*	0.94 (0.89 to 0.99)*
	Cumulative MDS	0.95 (0.90 to 0.99)*	0.97 (0.92 to 1.02)	0.97 (0.92 to 1.02)	0.98 (0.93 to 1.03)

HR=Hazard ratio per SD difference in each exposure; CI=Confidence interval.
Sample size = 9,349; person years = 149,681
Covariates in Models:
Model 1: sex, age.
Model 2: variables in Model 1 + education, employment, and time updated variables for smoking.
Model 3: variables in Model 2 + family history of myocardial infarction, family history of diabetes mellitus, time updated variables for prevalent diabetes mellitus, prevalent cancer, prevalent cardiovascular diseases, and anti-hypertensive medication.
Model 4: variables in Model 3 + time updated variables for BMI, SBP, DBP, TG, LDL, HDL, and total energy intake.
†Level of statistical significance: p-value ≥ 0.05, unless indicated by asterisk.
* P-value < 0.05
** P-value < 0.01
*** P-value < 0.001
†† SD increment in cumulative PAEE equals to 3.96 kJ/kg/day, and in cumulative MDS equals to 1.16 points,
Cumulative exposures are calculated by averaging MDS and PAEE at baseline and repeated assessments.

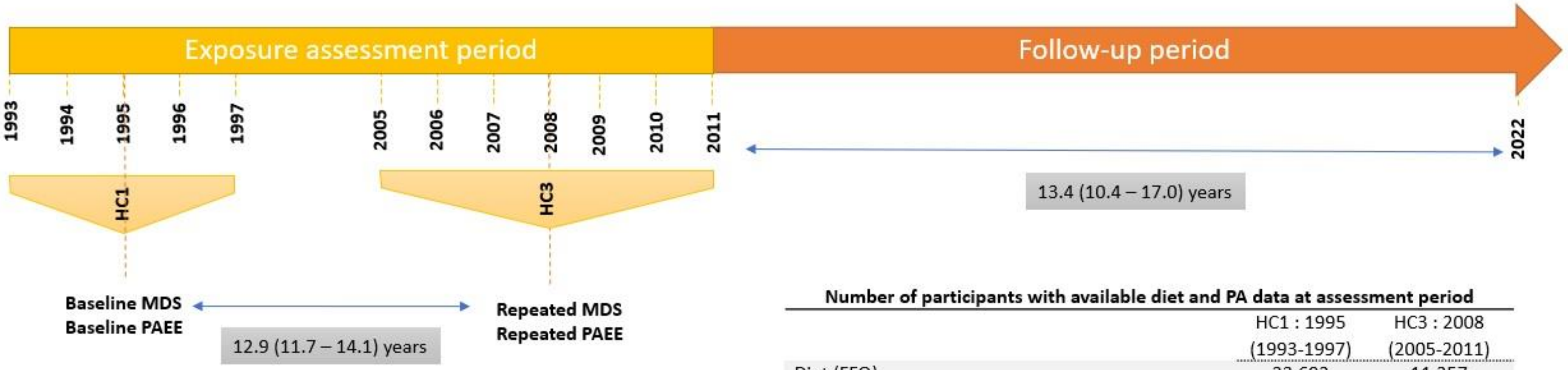

Number of participants with available diet and PA data at assessment period

	HC1 : 1995 (1993-1997)	HC3 : 2008 (2005-2011)
Diet (FFQ)	23,692	11,257
PA (short-EPIC questionnaire)	25,618	6,149

Different combinations of available diet and PA variables across assessments

Repeated diet (HC1 and HC3)	11,257
Repeated PA (HC1 and HC3)	5,910
Repeated diet and repeated PA	5,879

Figure S2 - EPIC-Norfolk Study design and timeline, considering health check 3 as the end of assessment period.
MDS=Mediterranean diet score; PAEE=physical activity energy expenditure; N/A= Not available; HC=health check; FFQ= food frequency questionnaire. Numbers in brackets above below arrow symbol indicate interquartile range of the years passed.

Table S7 - Associations of mutually adjusted exposures with mortality in the EPIC-Norfolk Study, considering health check 3 as the end of the assessment period†

Outcome	Exposures††	Model 1<br>HR (95% CI)	Model 2<br>HR (95% CI)	Model 3<br>HR (95% CI)	Model 4<br>HR (95% CI)
**All-cause mortality (1,726 deaths)**	Baseline PAEE	0.89 (0.83 to 0.95)***	0.90 (0.84 to 0.96)**	0.92 (0.86 to 0.98)*	0.91 (0.85 to 0.98)**
	ΔPAEE	0.89 (0.83 to 0.95)***	0.89 (0.85 to 0.96)**	0.91 (0.85 to 0.98)**	0.91 (0.85 to 0.98)**
	Baseline MDS	0.95 (0.90 to 1.00)	0.96 (0.91 to 1.01)	0.96 (0.91 to 1.01)	0.97 (0.92 to 1.03)
	ΔMDS	0.95 (0.90 to 1.00)	0.95 (0.90 to 1.00)	0.96 (0.90 to 1.01)	0.96 (0.91 to 1.01)
**CVD mortality (1,502 deaths)**	Baseline PAEE	0.87 (0.81 to 0.94)***	0.88 (0.82 to 0.95)**	0.91 (0.84 to 0.98)*	0.91 (0.84 to 0.98)*
	ΔPAEE	0.86 (0.80 to 0.93)***	0.87 (0.81 to 0.94)***	0.89 (0.83 to 0.96)**	0.89 (0.83 to 0.96)**
	Baseline MDS	0.96 (0.91 to 1.02)	0.97 (0.92 to 1.03)	0.97 (0.91 to 1.03)	0.98 (0.93 to 1.04)
	ΔMDS	0.95 (0.90 to 1.01)	0.95 (0.90 to 1.01)	0.96 (0.91 to 1.01)	0.96 (0.91 to 1.02)
**Cancer mortality (912 deaths)**	Baseline PAEE	0.89 (0.81 to 0.97)*	0.90 (0.82 to 0.99)*	0.92 (0.84 to 1.01)	0.92 (0.83 to 1.01)
	ΔPAEE	0.84 (0.77 to 0.93)***	0.85 (0.78 to 0.94)***	0.89 (0.81 to 0.97)*	0.88 (0.80 to 0.97)*
	Baseline MDS	0.98 (0.92 to 1.06)	1.00 (0.93 to 1.08)	1.01 (0.94 to 1.09)	1.02 (0.95 to 1.11)
	ΔMDS	0.95 (0.89 to 1.02)	0.96 (0.89 to 1.03)	0.97 (0.90 to 1.04)	0.96 (0.90 to 1.03)

HR=Hazard ratio per SD difference in each exposure; CI=Confidence interval.

Sample size = 5,878; person years = 69,456

Covariates in Models:

Model 1: sex, age.

Model 2: variables in Model 1 + education, employment, and time updated variables for smoking.

Model 3: variables in Model 2 + family history of myocardial infarction, family history of diabetes mellitus, time updated variables for prevalent diabetes mellitus, prevalent cancer, prevalent cardiovascular diseases, and anti-hypertensive medication.

Model 4: variables in Model 3 + time updated variables for BMI, SBP, DBP, TG, LDL, HDL, and total energy intake.

† Level of statistical significance: p-value ≥ 0.05, unless indicated by asterisk.

* P-value < 0.05

** P-value < 0.01

*** P-value < 0.001

†† SD increment in baseline PAEE equals to 4.64 kJ/kg/day, in ΔPAEE equals to 0.40 kJ/kg/day per year, in baseline MDS equals to 1.30 points, and in ΔMDS equals to 0.06 points per year.

Table S8 - Associations of mutually adjusted exposures with mortality in the EPIC-Norfolk Study, excluding deaths that occurred within two years of the last measurement †

Outcome	Exposures††	Model 1<br>HR (95% CI)	Model 2<br>HR (95% CI)	Model 3<br>HR (95% CI)	Model 4<br>HR (95% CI)
**All-cause mortality (3,392 deaths)**	Baseline PAEE	0.87 (0.83 to 0.91)***	0.88 (0.84 to 0.92)***	0.90 (0.86 to 0.94)***	0.90 (0.86 to 0.94)***
	ΔPAEE	0.88 (0.84 to 0.92)***	0.88 (0.85 to 0.92)***	0.90 (0.86 to 0.94)***	0.90 (0.86 to 0.94)***
	Baseline MDS	0.92 (0.89 to 0.96)***	0.94 (0.90 to 0.98)**	0.94 (0.90 to 0.98)**	0.95 (0.92 to 0.99)*
	ΔMDS	0.92 (0.88 to 0.96)***	0.93 (0.90 to 0.97)***	0.93 (0.89 to 0.97)***	0.93 (0.89 to 0.97)***
**CVD mortality (2,881 deaths)**	Baseline PAEE	0.87 (0.83 to 0.91)***	0.88 (0.84 to 0.92)***	0.91 (0.87 to 0.96)***	0.91 (0.87 to 0.95)***
	ΔPAEE	0.88 (0.84 to 0.92)***	0.89 (0.85 to 0.93)***	0.91 (0.86 to 0.95)***	0.91 (0.87 to 0.95)***
	Baseline MDS	0.91 (0.88 to 0.95)***	0.93 (0.89 to 0.97)**	0.93 (0.89 to 0.97)**	0.95 (0.91 to 0.99)*
	ΔMDS	0.92 (0.88 to 0.96)***	0.93 (0.89 to 0.97)***	0.93 (0.89 to 0.97)**	0.93 (0.89 to 0.97)**
**Cancer mortality (1,712 deaths)**	Baseline PAEE	0.89 (0.84 to 0.95)***	0.90 (0.85 to 0.96)**	0.92 (0.87 to 0.98)*	0.92 (0.87 to 0.98)**
	ΔPAEE	0.89 (0.83 to 0.93)***	0.90 (0.84 to 0.95)***	0.92 (0.86 to 0.97)**	0.91 (0.86 to 0.97)**
	Baseline MDS	0.95 (0.90 to 1.00)	0.98 (0.92 to 1.03)	0.97 (0.92 to 1.03)	0.99 (0.93 to 1.05)
	ΔMDS	0.93 (0.88 to 0.98)**	0.94 (0.89 to 1.00)*	0.94 (0.89 to 1.00)*	0.94 (0.89 to 0.99)*

HR=Hazard ratio per SD difference in each exposure; CI=Confidence interval.

Sample size = 9,205; person years = 149,499

Covariates in Models:

Model 1: sex, age.

Model 2: variables in Model 1 + education, employment, and time updated variables for smoking.

Model 3: variables in Model 2 + family history of myocardial infarction, family history of diabetes mellitus, time updated variables for prevalent diabetes mellitus, prevalent cancer, prevalent cardiovascular diseases, and anti-hypertensive medication.

Model 4: variables in Model 3 + time updated variables for BMI, SBP, DBP, TG, LDL, HDL, and total energy intake.

† Level of statistical significance: p-value ≥ 0.05, unless indicated by asterisk.

* P-value < 0.05

** P-value < 0.01

*** P-value < 0.001

†† SD increment in baseline PAEE equals to 4.64 kJ/kg/day, in ΔPAEE equals to 0.65 kJ/kg/day per year, in baseline MDS equals to 1.30 points, and in ΔMDS equals to 0.33 points per year.

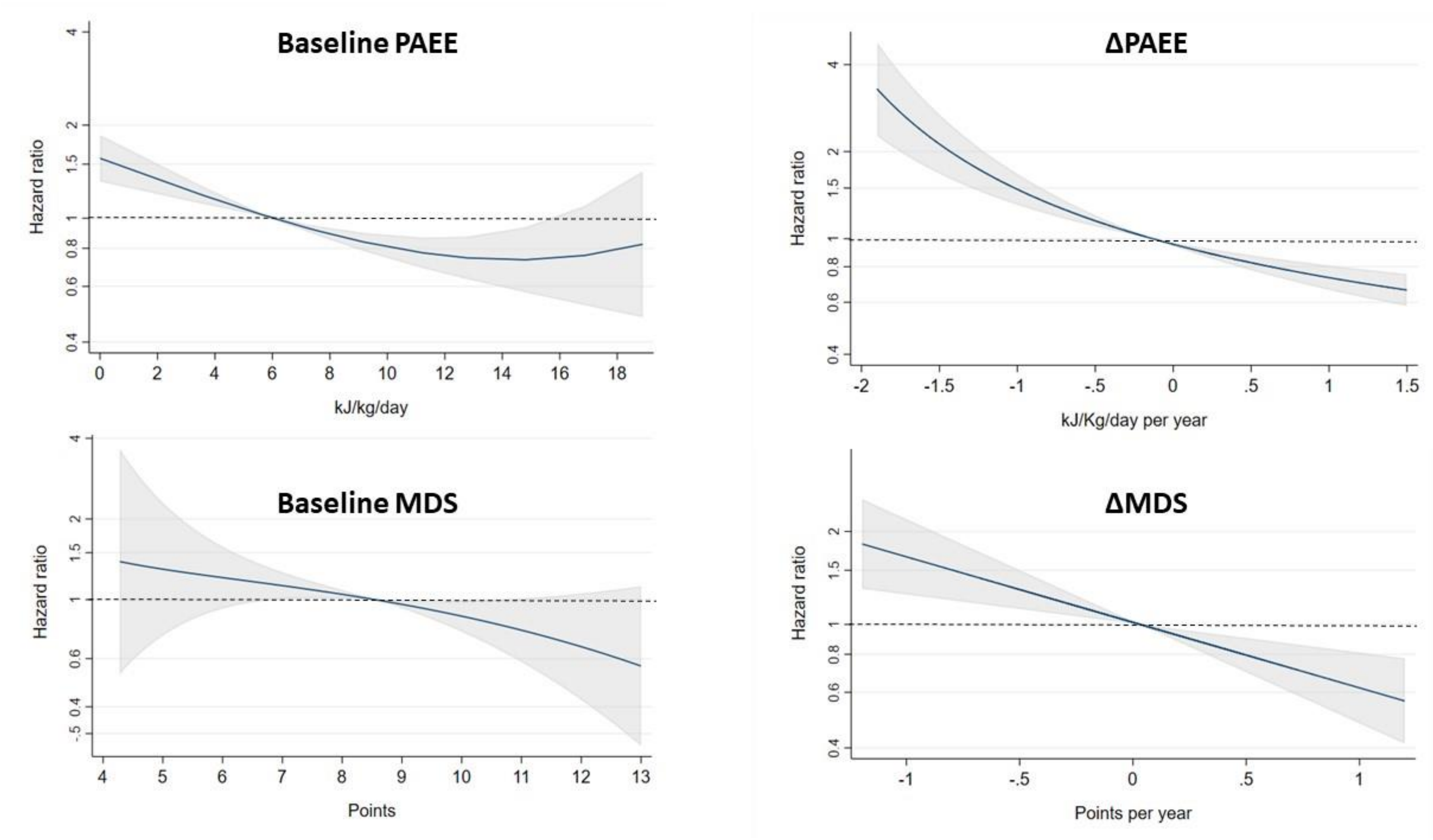

Figure S3 - Dose-response relationship between PA and diet exposures and all-cause mortality fitted by using a Cox Proportional-Hazards with fractional polynomial in the EPIC-Norfolk Study.
Model 4 was used for this analysis (see methods).
The 95% CI is depicted in the shaded regions.
MDS=Mediterranean diet score; PAEE=physical activity energy expenditure; ΔMDS=over time changes in MDS; ΔPAEE=over time changes in PAEE.

A

Repeated PAEE level	Baseline PAEE level: Low<br>0	Medium<br>5.3 (1.7)	High<br>11.6 (2.7)
**High**<br>**11.7 (2.7)**	0.83 (0.60 to 1.15)<br>39 death / 1,950 pys	0.74 (0.63 to 0.87)<br>205 death / 12,876 pys	0.73 (0.64 to 0.85)<br>277 death / 22,616 pys
**Medium**<br>**5.2 (1.7)**	0.81 (0.70 to 0.94)<br>213 death / 10,385 pys	0.80 (0.73 to 0.89)<br>753 death / 37,214 pys	0.79 (0.70 to 0.89)<br>378 death / 19,957 pys
**Low**<br>**0**	1.00 (ref)<br>791 death/ 19,481 pys	0.95 (0.86 to 1.06)<br>689 death / 19,449 pys	1.04 (0.89 to 1.22)<br>189 death / 5,753 pys

B

Repeated MDS level	Baseline MDS level: Low<br>7.1 (0.7)	Medium<br>8.5 (0.3)	High<br>10.0 (0.7)
**High**<br>**10.1 (0.6)**	0.90 (0.73 to 1.10)<br>108 death / 4,870 pys	0.85 (0.74 to 0.97)<br>303 death / 14,977 pys	0.86 (0.77 to 0.97)<br>581 death / 31,421 pys
**Medium**<br>**9.7 (0.3)**	0.87 (0.76 to 0.98)<br>352 death / 14,917 pys	0.82 (0.73 to 0.93)<br>463 death / 20,375 pys	1.0 (0.88 to 1.14)<br>338 death / 14,770 pys
**Low**<br>**7.3 (0.7)**	1.00 (ref)<br>837 death / 29,409 pys	1.04 (0.93 to 1.18)<br>421 death / 14,302 pys	1.02 (0.85 to 1.24)<br>131 death / 4,640 pys

C

ΔMDS	ΔPAEE: Decreasers<br>≤ -0.1	Maintainers<br>-0.1 to 0.1	Increasers<br>≥ 0.1
**Increasers**<br>**≥ 0.1**	0.83 (0.73 to 0.94)<br>108 death / 4,870 pys	0.78 (0.68 to 0.89)<br>303 death / 14,977 pys	0.69 (0.59 to 0.81)<br>581 death / 31,421 pys
**Maintainers**<br>**0.1 to 0.1**	0.91 (0.80 to 1.04)<br>352 death / 14,917 pys	0.84 (0.73 to 0.97)<br>463 death / 20,375 pys	0.74 (0.62 to 0.89)<br>338 death / 14,770 pys
**Decreasers**<br>**≤ -0.1**	1.00 (ref)<br>837 death / 29,410 pys	0.90 (0.79 to 1.03)<br>421 death / 14,302 pys	0.67 (0.57 to 0.80)<br>131 death / 4,640 pys

Figure S4 - Associations of different trajectories of PA and diet with all-cause mortality in the EPIC-Norfolk Study, based on three-by-three levels of exposures

Analyses are based on Model 4 (see methods). MDS=Mediterranean diet score; PAEE=physical activity energy expenditure; ΔMDS=over time changes in MDS; ΔPAEE=over time changes in PAEE.

A) Model mutually adjusted for baseline and repeated MDS. Cut offs for PAEE: Low PAEE = no PA reported, Medium PAEE = below 10 kJ/kg/day (WHO recommendations for additional health benefits), High PAEE = equal or above 10 kJ/kg/day.

B) Model mutually adjusted for baseline and repeated PAEE. Cut offs for MDS: Low MDS = 1st Tertile of MDS in population (< 8 points), Medium MDS 2nd Tertile of MDS in population (≥ 8 and ≤ 9.1 points), High MDS = 3rd Tertile of MDS in population (> 9.1 points).

C) Model mutually adjusted for baseline PAEE and baseline MDS. ΔPAEE is expressed in kJ/kg/day per year, ΔMDS is expressed in points per year.

Table S9- Population impact by estimating the differences in total number of deaths that could have been potentially observed under two different counterfactual scenarios.

**Trajectory Group (% of total sample)**[†]	**Scenario under observed exposures**			**Counterfactual scenario 1** Applying the adjusted mortality rate of G1 to all groups			**Counterfactual scenario 2** Applying the adjusted mortality rate of G16 to all groups		
	Crude deaths	Adjusted mortality rate[‡]	Adjusted number of deaths	Number of deaths expected	Difference from the number of deaths observed	Percentage of contribution to total difference	Number of deaths expected	Difference from the number of deaths observed	Percentage of contribution to total difference
G1 (10.4%)	522	3,095	433.9	433.9	Ref	Ref	369.8	-64	19%
G2 (3.0%)	101	2,943	120.5	125.2	+5	2%	106.7	-14	4%
G3 (5.1%)	219	2,852	198.0	210.7	+13	5%	179.5	-18	5%
G4 (1.6%)	44	2,119	50.7	68.2	+18	6%	58.1	+7	-2%
G5 (7.2%)	357	3,443	327.3	302.9	-24	-9%	258.3	-69	20%
G6 (11.7%)	330	2,762	449.5	490.0	+41	15%	417.8	-32	9%
G7 (3.7%)	150	2,813	144.1	154.9	+11	4%	132.1	-12	3%
G8 (5.7%)	134	2,606	207.6	236.7	+29	11%	201.8	-6	2%
G9 (4.1%)	199	3,260	177.3	170.6	-7	-2%	145.4	-32	9%
G10 (1.4%)	44	3,042	55.7	56.4	+1	0%	48.1	-8	2%
G11 (13.1%)	475	2,775	499.5	542.7	+43	16%	462.5	-37	11%
G12 (4.3%)	114	2,461	150.2	179.2	+29	11%	152.7	+3	-1%
G13 (2.5%)	129	3,472	115.2	106.0	-9	-3%	90.4	-25	7%
G14 (4.2%)	119	2,774	163.3	177.4	+14	5%	151.2	-12	3%
G15 (7.8%)	295	2,831	305.4	326.6	+21	8%	278.5	-27	8%
G16 (14.2%)	302	2,514	506.3	594.0	+88	32%	506.3	Ref	Ref
Sum	3534		3904	4175	+271 (Standard Error: 127)§	100%	3559	-345 (Standard Error: 129)§	100%
**Percentage of difference in total deaths of counterfactual scenarios vs. observed adjusted deaths (95% CI)**				**+6.9% (0.6% to 13.3%)§**			**-8.8% (-15.3% to -2.4%)§**		

†G = group. See figure 2 for the definition of each trajectory group.

‡Adjusted mortality rate is expressed as deaths per 10^5 person-years and is based on covariates in the Model 4 (see methods).

§ Standard error of the count or percent points, estimated with a bootstrap technique.
